# Toward 3D-bioprinting of an endocrine pancreas: A building-block concept for bioartificial insulin-secreting tissue

**DOI:** 10.1177/20417314221091033

**Published:** 2022-04-20

**Authors:** Gabriel A Salg, Eric Poisel, Matthias Neulinger-Munoz, Jamina Gerhardus, Daniel Cebulla, Catrin Bludszuweit-Philipp, Vitor Vieira, Felix Nickel, Ingrid Herr, Andreas Blaeser, Nathalia A Giese, Thilo Hackert, Hannes G Kenngott

**Affiliations:** 1 Department of General, Visceral and Transplantation Surgery, University Hospital Heidelberg, Heidelberg, Germany; 2 Department of Dermatology and Allergy, University Hospital LMU Munich, Munich, Germany; 3 Technical University of Darmstadt, Institute for BioMedical Printing Technology, Darmstadt, Germany; 4 ASD Advanced Simulation and Design GmbH, Rostock, Germany; 5 INOVA DE GmbH, Heidelberg, Germany

**Keywords:** Bioprinting, tissue engineering, endocrine pancreas, next-generation sequencing, diabetes

## Abstract

Three-dimensional bioprinting of an endocrine pancreas is a promising future curative treatment for patients with insulin secretion deficiency. In this study, we present an end-to-end concept from the molecular to the macroscopic level. Building-blocks for a hybrid scaffold device of hydrogel and functionalized polycaprolactone were manufactured by 3D-(bio)printing. Pseudoislet formation from INS-1 cells after bioprinting resulted in a viable and proliferative experimental model. Transcriptomics showed an upregulation of proliferative and ß-cell-specific signaling cascades, downregulation of apoptotic pathways, overexpression of extracellular matrix proteins, and VEGF induced by pseudoislet formation and 3D-culture. Co-culture with endothelial cells created a natural cellular niche with enhanced insulin secretion after glucose stimulation. Survival and function of pseudoislets after explantation and extensive scaffold vascularization of both hydrogel and heparinized polycaprolactone were demonstrated *in vivo*. Computer simulations of oxygen, glucose and insulin flows were used to evaluate scaffold architectures and Langerhans islets at a future perivascular transplantation site.

## Introduction

Transplantation of islets of Langerhans to selected patients with type 1 and type 3c diabetes mellitus (DM) is an established treatment option.^1,2^ Autologous transplantation can be performed after isolation of islets from the resected pancreas without the need for life-long immunosuppression, whereas islet transplants for patients with type 1 DM rely on allogenic donor islets.^2^ Current therapeutic limitations include a shortage of donor material but also a substantial loss of islets and impaired long-term function post transplantation.^1,3–5^ Scaffold-based tissue engineering approaches extend the range of possible transplantation sites and might present a long-term curative treatment.^3,4,6–9^ For a successful translational approach, several requirements and properties of a functional tissue-engineered device have to be considered. The scaffold material itself should not induce cytotoxicity or extensive foreign body response and should preferably support or promote rapid vascularization.^10,11^ Furthermore, the scaffold should be retrievable and possess a certain mechanical strength, at least until tissue remodeling has occured.^10,11^

Previously, we found that scaffold-based tissue engineering is still hampered by reduced vascularization, causing insufficient nutrition, hypoxia, and immunological host-graft reactions.^3^ The multitude of studies focusing mostly on aspects of the tissue engineering network have not yet provided structured evidence to define a gold-standard approach. Investigations of a variety of different cells, scaffold materials, fabrication techniques, and transplantation sites have not yet consolidated into an entire process leading toward bioartificial organs. In an integrated, multilevel approach, tissue-engineered building-blocks are investigated on a molecular level against the background of a macroscopic device for translation to further steps. The end-to-end concept presented here aims to address the challenges of hybrid scaffold fabrication, cellular integration, and functional evaluation to provide experimental proof of function for a 3D-bioprinted hybrid scaffold for insulin-secreting cells that is designed for clinical application.

## Materials and methods

### Computer-aided design (CAD) model creation and slicing for hybrid scaffold fabrication

CAD models of the scaffold structures were created using the open source package Blender.^12^ The models were converted from Standard Triangulation Language (STL) to numerical control G programming language using the Cura software package (v4.1, Ultimaker, Utrecht, NL; available from https://www.ultimaker.com/en/products/ultimaker-cura-software) for dual-extrusion 3D printing of the polycaprolactone (PCL) outer shell. For hydrogel 3D bioprinting an integrated slicing software was applied (CellInk, Gothenburg, Sweden).

### 3D-printing of PCL and heparin surface functionalization for hybrid scaffold

PCL components were fabricated using a dual-extrusion-based 3D printer (UM S5, Ultimaker, Utrecht, Netherlands). Polycaprolactone (PCL) filament (Facilan^TM^ PCL 100 Filament 2.85 mm, 3D4Makers, Haarlem, Netherlands; MW: 50,000 g/mol) was used for the scaffold structure and polyvinyl alcohol filament (PVA; Ultimaker) as a sacrificial, water-soluble support structure. PCL was extruded with an AA 0.25 mm, PVA with a BB 0.4 mm print head, using the following settings: print speed 20 mm/s, build plate temperature 30°C, fan speed 100%, AA print head temperature 140°C, BB print head temperature 215°C. In order to print PCL at temperatures as low as 140°C, the g-code was manually edited by prefix code “M302” to avoid device-specific conformity checks. For heparin surface functionalization, 1% (w/v) heparin (Lot# H0200000, Merck, Darmstadt, Germany) was dissolved in 0.05 m 2-(N-morpholino) ethanesulfonic acid monohydrate (MES) buffer (Lot# K49565026903, Merck) at a pH of 5.5. Quantities of 0.5 m 1-ethyl-3-(3-dimethylaminopropyl) carbodiimide (EDC) (Lot# E7750, Merck) and 0.5 m N-hydroxysuccinimide (NHS) (Lot# BCBW6640, Merck) were added to the heparin solution. Scaffolds were previously equilibrated for 30 min in MES buffer and subsequently immersed in reaction mixture. The reaction mixture was then stirred for 8 h at room temperature. The reaction was stopped by extensive washing with sterile H_2_O to remove unbound heparin. Scaffolds were stored in PBS. Dried PCL scaffolds and heparin-coated PCL scaffolds were 10 nm gold/platinum sputtered (Leica EM ACE 600, Leica Microsystems GmbH, Wetzlar, Germany) and qualitatively analyzed by scanning electron microscopy (Zeiss Leo Gemini 1530, Carl Zeiss AG, Oberkochen, Germany). Images were taken at different magnifications with an accelerating voltage of 2.0 kV.

### Cell culture

The rat INS-1 832/3 cell line (referred to as INS-1 hereinafter) was obtained from Merck (Darmstadt, Germany). A HUVEC cell line was obtained from the American Type Culture Collection (Manassas, VA, USA). Mycoplasma testing was performed monthly by polymerase chain reaction. INS-1 cells were used until passage 10; insulin-producing function was ensured by selection through Geneticin resistance. INS-1 cells were cultivated in RPMI-1640 (Gibco, Thermo Fisher Scientific, Waltham, MA, USA) supplemented with 10% fetal bovine serum (Gibco), 1% Geneticin (Merck), 1% HEPES 1 M (Gibco), 1% sodium pyruvate 100 mM (Merck), and 0.1% 2-mercaptoethanol (Merck). HUVEC cells were cultivated in endothelial cell growth medium (Lot# 211–500, Cell Applications, San Diego, CA, USA) supplemented with 1% penicillin/streptomycin (Merck) and 5% fetal bovine serum. For co-cultures, culture medium composition was chosen according to cell ratio. Cells were grown in T75 flasks (Falcon®, Corning, NY, USA) at 37°C and 5% CO_2_.

### Bioprinting of cell-laden hydrogels for hybrid scaffold

Bioprinting was performed using the BioX from CellInk. Pneumatic extrusion print heads were used for extrusion of bioink. 3 × 10^6^ cells/ml hydrogel were used for bioprinting. The cells, either INS-1 only or INS-1 with HUVEC cells in 1:2 ratio, were diluted in either RPMI-1640 or a 1:2 mixture of RPMI-1640 and endothelial cell growth medium and gently mixed 1:10 with GelXA LAMININK-411 hydrogel (Lot# IK-3X2123, CellInk) using female-female Luer-lock-adapted syringes. The INS-1/HUVEC ratio was chosen based on the natural islet microenvironment^5^ and due to superior results, compared with a 1:5 ratio. The cell-laden hydrogel was transferred to a UV-shielded cartridge and centrifuged at 100*g* for 1 min to remove any air. The cartridge (pre-cooled to 4°C) was loaded into pneumatic print heads. Bioprinting in 24-well plates (Falcon®, Corning) was performed with the following settings for proliferation assays, CAM xenotransplantation, and glucose-stimulated insulin secretion (GSIS): droplet print mode, 2.6 s extrusion time, 30 kPa extrusion pressure, 2 s ultraviolet (UV) crosslinking (405 nm) at 5 cm distance. After printing, the hydrogel domes were incubated in 1 ml of either RPMI-1640 (INS-1 only) or in a 1:2 mixture RPMI-1640 and endothelial cell growth medium (co-culture). Grid-like structures were printed in 24-well plates to perform metabolic assays and total RNA isolation using a 21-gauge conical nozzle, extrusion pressure 23 kPa, print speed 8 mm/s, 50 ms pre-flow delay, infill 15%, 2 s crosslinking at 405 nm with 5 cm distance to printed layer.

### Investigation of crosslinking-dependent porosity: Scanning electron microscopy and diffusion assay of bioprinted hydrogel structures

Specimen were prepared by mixing PBS 1:10 with GelXA LAMININK-411 hydrogel. 300 µl were transferred into 48-well plates. UV crosslinking of 2 s, 5 s, 10 s, respectively, was performed at 405 nm, 5 cm distance (145mW/cm^2^) using the OmniCure S2000 UV-curing system (Excelitas Technologies, Waltham, MA, USA). Cured samples were frozen in liquid nitrogen and freeze dried using the Alpha 1–2 LDplus freeze drying system (Martin Christ Gefriertrocknungsanlagen GmbH, Osterode, Germany). Samples were kept under vacuum at −55 °C for 22 h for drying. Scanning electron microscopy specimen preparation was equal to PCL scaffold investigation. For diffusion assays specimen were prepared by mixing PBS with hydrogel 1:10. The mixture was loaded into channels (75% filling) of µ-Slides VI 0.4 (Lot# 190322/7, Ibidi, Graefelfing, Germany) mounted on microscope slides. Crosslinking was performed using the BioX bioprinter and its 405 nm UV module at 5 cm distance. Groups of 2 s, 5 s, and 10 s crosslinking time were investigated. About 50 µg/ml FITC-Dextran (Lot# BCCD6370, Sigma-Aldrich) was filled into channels and the lid was closed to prevent drying of samples. Diffusion of FITC-Dextran into the gel was monitored at set timepoints for 2 h using the ECHO Revolve microscope. Images were analyzed using the plot profile in Fiji package of imageJ.^13^

### Detection of metabolic activity, proliferation, and apoptosis

For a visual assessment of metabolic activity, INS-1 cells in bioprinted grid scaffolds were stained with thiazolyl blue tetrazolium bromide (MTT, Merck) after 5 days (or 7 days, respectively) in culture. A quantity of 100 μL of 5 mg/ml MTT dissolved in PBS was added to 900 μL INS-1 culture medium and scaffolds were incubated under cell culture conditions for 2 h. Images were taken using a Leica DMi8 fluorescent microscope. Viability and proliferation of the bioprinted, encapsulated INS-1 cells were determined using an ATP-based assay with luminescent readout (CTG, CellTiter-Glo® 3D Cell Viability Assay, Promega GmbH, Walldorf, Germany) according to the manufacturer’s protocol. In brief, droplets printed in 96-well plates were incubated with 100 µl INS-1 expansion medium. On days 0, 3, 6, 9, and 12 after printing, blinded sample droplets together with 100 µl medium were transferred into a 96-well solid white polystyrene microplate (Falcon®, Corning) and 100 µl CTG reagent was added to each well. The microplate was continuously shaken for 25 min, and luminescence was measured using an ELISA reader (Synergy HTX, multi-mode reader, BioTek, Bad Friedrichshall, Germany). For apoptotic cell detection, fixated bioprinted grid-structures including encapsulated INS-1 cells (day 12 post-printing) were embedded using HistoGel™ (Lot# 370,234, HG-4000-012, Thermo Fisher Scientific) and cryomolds (Tissue-Tek™, Cryomold™, Thermo Fisher Scientific) according to the manufacturers’ instructions. Apoptotic cells were detected using cleaved Caspase-3 immunohistochemical staining as described previously.^14,15^ Staining of paraffin embedded tissue was performed after antigen retrieval at pH 9.0 using a polyclonal rabbit cleaved caspase 3 antibody (overnight 1:300 in background reducing agent, Asp 175, Lot#47, 96611S, Cell Signaling Technology, Boston, MA, USA) and counter-staining with hematoxylin.

### Live-cell imaging for migration tracking

Imaging was performed using brightfield microscopy (BZ-X800 fluorescent microscope, Keyence, Neu-Isenburg, Germany) and a stage top incubator (WSKMX, Tokai Hit, Japan) with a constant temperature of 37.5°C, 5% CO_2_ level and constant humidity. Bioprinted INS-1 containing grids were investigated on day 3 post-printing. Live-cell imaging was conducted at 328 consecutive time points with an interval of 5 min. At each time point z-stack imaging was performed (74 stacks, 2.2 µm pitch, 1/80 s exposure time, gain + 6dB, monochrome 8bit, no binning). Image processing was performed using the BZ analyzer software (12/2021). Full focus images were created from z-stacks for each time point. Manual cell tracking on the imaging sequence was performed using the TrackMate plugin^16^ on ImageJ.

### RNA sequencing

Genome-wide expression profiling was a service provided by the European Molecular Biology Laboratory (EMBL; Heidelberg, Germany). After 5 days in culture total RNA was isolated from 2D monolayer culture and 3D hydrogel culture using a RNeasy Mini kit (Qiagen, Hilden, Germany) according to the manufacturer’s instructions (biological replicates, passage 3). After isolation, the total RNA was treated with the Turbo DNAfree kit according to the manufacturer’s instructions (Thermo Fisher Scientific). The RNA concentration and quality were evaluated using Nanodrop and Agilent2000 Bioanalyzer. RNAseq libraries were prepared using the TruSeq stranded mRNA kit and sequenced using an Illumina NextSeq 500 platform, resulting in 75-bp single end reads in a read count of 36 million reads per sample. Quality control of the RNAseq FastQ files was performed with FastQC v.0.11.8. The obtained reads were pseudoaligned using the rn4 reference genome with the addition of human insulin gene and quantified by Salmon v1.2 with standard parameters. The resulting transcript expression levels were summarized to gene-level expression values and corrected for average transcript length by using tximport v1.10.1 and the “lengthScaledTPM” option while filtering out genes expressed in low amounts (average counts < 10).^17^ Differentially expressed genes for the culture conditions were determined by using the DESeq2 v1.22.2 package.^18^ Using the DESeq2 and log2 fold change pre-ranked differentially expressed genes, a gene set enrichment analysis was performed using the fgsea package v1.8 and the hallmark gene sets from MSigDB v7.1.^19^ Additional data analysis was performed using Ingenuity Pathway Analysis (IPA; Ingenuity Systems, Qiagen) by input of gene identifiers, log2 fold change, and *p*-values.^20^ Canonical pathway analysis identified the pathways referenced in the Ingenuity Knowledge Base of canonical pathways (11/2020) that were significant to the data set (*p* < 0.05).

### Xenotransplantation to the chorioallantoic membrane (CAM) of fertilized chicken eggs

As described before,^21^ fertilized eggs from genetically identical hybrid Lohman Brown chickens were obtained from a local ecological hatchery (Gefluegelzucht Hockenberger, Eppingen, Germany). Eggs were delivered at day 0 of chick development and were immediately cleaned with 70% warm ethanol. The eggs were placed in a digital motor breeder (Type 168/D, Siepmann GmbH, Herdecke, Germany) at 37.8°C and 45–55% humidity with an activated turning mechanism to start day 1 of the embryonic chick development. 4 days after incubation, the turning mechanism of the incubator was switched off and a small hole was cut into the eggshell to detach the embryonic structures from the eggshell by removing 3 ml albumin. The hole was covered with Leukosilk® tape (BSN medical, Hamburg, Germany), and the eggs were incubated further with the turning mechanism switched off. On day 9 of embryonic development, the tape was removed and the epithelial layer of the chorioallantoic membrane (CAM) was gently scratched with a syringe needle to ensure immediate blood supply to the xenotransplant/polymer component. PCL scaffold groups and bioprinted xenografts (bioprinted hydrogel) were placed on the CAM. PCL scaffold groups consisted of 3D-printed PCL scaffolds functionalized with covalently bound heparin and plain PCL scaffolds. Prior to implantation, scaffolds were sterilized with 70% ethanol for 48 h. For explantation, the chicks were ethically euthanized at day 18 of development, 3 days before hatching, as described before.^22^ PCL scaffolds and bioprinted xenografts were excised including the surrounding CAM and briefly washed in PBS before further imaging. Each specimen was imaged by stereomicroscopy (Leica MZ10 F, Leica Microsystems GmbH, Wetzlar, Germany). Images of PCL scaffold groups were analyzed using an automatic image analysis software (WimCAM; CAM Assay Image Analysis Solution, Release 1.1, Wimasis, 2016).

### Immunohistochemistry of xenograft tissue

Xenografts were fixated in 5% formaldehyde (Otto Fischar GmbH & Co. KG, Saarbruecken, Germany) after excision and transferred to 70% ethanol after 24 h. The fixated, explanted xenografts were embedded using HistoGel™ (Lot# 370234, HG-4000-012, Thermo Fisher Scientific) and cryomolds (Tissue-Tek™, Cryomold™, Thermo Fisher Scientific) according to the manufacturers’ instructions. After paraffin embedding of the xenografts, randomly chosen blocks from each group were continuously sampled in 5 µm serial sections, numbered, and processed for histology. Slides with odd numbers were stained with Mayer’s Hematoxylin-Eosin (H/E), while those with even numbers were immunostained for insulin. Therefore, a primary insulin antibody (monoclonal mouse IgG, 2D11-H5, Lot# SC-8033, SantaCruz, Dallas, TX, USA), overnight 1:100 in background reducing antibody diluent (S3022, Dako, Agilent Tech., Santa Clara, CA, USA), and a polyclonal goat anti-mouse secondary antibody (Dako, Agilent Tech.), 3-3′diaminobenzidine staining with subsequent hematoxylin counter-staining, were used. Further samples were immunostained for endothelial and endothelial progenitor cells with a primary chicken CD34 antibody (monoclonal mouse IgG; Lot# AV138, UniProt E1BUT3, Avian Immunology toolbox project, Bio-Rad Laboratories GmbH, Feldkirchen, Germany) to identify newly formed vascular structures in the CAM assay. For final validation of spatial relationships, tissue slices were double-stained using both a primary insulin (2D11-H5, Santa Cruz) and CD-31 antibodies (overnight 1:250 in background reducing agent, monoclonal rabbit IgG; Lot# EPR17259, ab182981, Abcam, Cambridge, UK ) using 3-3′diaminobenzidine staining for insulin, a biotin-streptavidin system for CD31 staining (Vector® BA-1000, goat anti-rabbit secondary IgG; Lot# ZH0818; IgG, and Vector® Red Substrate Kit SK-5100, Lot# Reagent1 ZH0305, Reagent2 ZH0308, Reagent3 ZH0305 Vector Laboratories Inc., Burlingame, CA, USA) and subsequent hematoxylin counter-staining. Target retrieval was performed at both pH 6.0 and pH 9.0 for insulin and CD31 antibody, respectively. Whole slides were scanned at 40× magnification using a NanoZoomer S60 Digital Slide Scanner (Hamamatsu Photonics, Hamamatsu City, Japan). Insulin-stained tissue slides were analyzed using the ilastik software package^23^ for supervised machine learning (ilastik: interactive machine learning for [bio]image analysis, v1.3.3, open-source, https://www.ilastik.org/download.html). Pseudoislets (insulin mono-staining) were segmented using the pixel classification workflow (islet, non-islet, background [not islet, not non-islet]). First, a random forest classifier was trained manually, and subsequent batch processing was performed. Due to limitations of the machine-learning strategy in differentiating xenograft and CAM tissue, the xenograft area was determined manually using ImageJ (Fiji package^13^).

### Glucose-stimulated insulin secretion (GSIS)

For GSIS experiments, INS-1 cells were stained with red fluorescent membrane inserting dye PKH-26 (Lot# SLBW0232, Merck) according to the manufacturer’s protocol prior to mixing with the hydrogel for bioprinting. In brief, cells were trypsinized using 0.25% Trypsin-EDTA (Gibco), rinsed with Dulbecco’s PBS (DPBS; PromoCell GmbH, Heidelberg, Germany), and finally pelleted. The pellet was resuspended in Diluent A, and PKH-26 dye dissolved in Diluent A was added to the cells. After rapid mixing and incubation, culture medium was added. The cell suspension was centrifuged and further washing steps were performed. The insulin secretion of 3D-bioprinted INS-1 (low glucose: *n* = 22; high glucose: *n* = 20) and INS-1/HUVEC co-culture (low glucose: *n* = 22; high glucose: *n* = 21) groups, INS-1 cells seeded on PCL/HEP-PCL scaffolds (2 × 10^5^ cells in 1 ml RPMI-1640 per well), and the 2D monolayer control group was measured. In the 2D monolayer culture group, INS-1 cells were seeded in 4-well chamber slides (10^5^ cells in 1 ml RPMI-1640 per well) (Nunc® Lab-Tek®, Thermo Fisher Scientific). The medium was changed after 2 days, and GSIS was performed on day 3 in all conditions. In case of medium change and experimental execution of GSIS on PCL/HEP-PCL scaffolds, scaffolds were simply removed from cultivation medium, gently rinsed with PBS, and placed into new medium-containing wells to avoid bias from cells growing attached to the culture plate. For preparation of the GSIS solution, SILAC RPMI-1640 Flex (A2494201, Gibco) was supplemented with MgSO_4_ (1.16 mmol/l end concentration) (Merck), CaCl_2_ (2.5 mmol/l end concentration) (Merck), 20 mM HEPES, and 0.2% BSA (Merck). GSIS was initiated by rinsing the cells once with low-glucose solution (1.67 mM D-glucose), followed by incubation for 1 h in 1 ml low-glucose solution. After that, either 1 ml of low-glucose solution or 1 ml of high glucose solution (16.7 mM D-glucose) was added, followed by incubation for 2 h. A quantity of 500 µl medium was taken and briefly spun down in a 1.5-ml Eppendorf tube. Next, 400 µl supernatant was used for determination of insulin concentration by chemiluminescence immunoassay (ADVIA CENTAUR, Siemens Medical Solutions, Malvern, PA, USA). After GSIS of 2D samples on chamber slides, cells were incubated in 5% formaldehyde solution for 15 min, rinsed with DPBS twice, dried for 10 min, and covered with Fluoroshield Mounting Medium with 4′,6-diamidino-2-phenylindole (DAPI; Abcam) and a coverslip. Similarly, 3D-bioprinted samples were fixated and transferred to a glass slide, covered with two drops of Shandon Consul mounting medium (Thermo Fisher Scientific), and squashed with a coverslip until flattened. Cells were counted using a Leica DMi8 fluorescence microscope with the following settings for PKH-26 imaging: 10× magnification, Y3 filter block, 260 ms exposure time, gain 7. DAPI imaging was performed with the following settings: 10× magnification, DAPI filter block, 10.5 ms exposure time, gain 4. Image processing was performed using Leica LAS × software, and subimages were assembled to mosaics depicting whole domes or whole well bottoms. Cells were counted using ImageJ (Fiji package^13^). In the case of polymer scaffold culture, cells were lysed using radioimmunoprecipitation assay (RIPA) buffer supplemented with protease inhibitor (cOmplete Mini, Roche, Basel, Switzerland) and incubated on ice for 10 min. Protein concentration was determined using a bicinchoninic acid (BCA) assay (Pierce BCA Protein Assay Kit, Thermo Fisher Scientific). The assay was performed according to the manufacturer’s protocol. In *n* = 12 wells of a 24-well plate, 10^5^ INS-1 cells were seeded in 1 ml RPMI-1640 for correlation of total protein to cell number. After 48 h the medium was changed, followed by another 24 h of incubation. Cells were lysed using 250 µl RIPA buffer and protease inhibitor in *n* = 6 wells and total protein was determined. The residual wells were fixed with 5% formalin, rinsed twice with PBS, and mounted using Fluoroshield Mounting Medium with DAPI. After cell counting, a conversion factor between cell number and total protein was obtained.

### Computer-aided applicability screening of scaffold architecture by finite element analysis

Diffusion of oxygen, glucose, and secreted insulin through islets of Langerhans encapsuled in a hydrogel shell was modeled using a custom python script (v3.8, Python Software Foundation, https://www.python.org) for input parameter-based insulin secretion based on literature data^24^ (Supplementary Figure 11). The finite element simulations are based on mesh generation using the open-source module Gmsh^25^ and FiPy,^26^ a respective finite element solver. The simulations were performed in 2D and the results were extrapolated to 3D spherical setups. Hydrogel shell and islet were initialized with 10 mM (5, 15, and 25 mM) glucose. The initial oxygen partial pressures ranged from 90 mmHg to 270 mmHg. The thickness of the hydrogel shell varied between 0 µm and 1000 µM. In the simulation, diffusion started from outside the capsule and triggered consumption of glucose and oxygen within the islets. The simulations were carried out for at least 60 s with step sizes for diffusion below 0.005 s leading to converged results. Based on simulation results, cell viability was evaluated by considering a minimum local oxygen partial pressure of 0.07 mmHg for cells to survive.

### Statistical analysis

Data analysis and statistical testing was done using R version 3.6.1 and the ggplot2 package. Proliferation assay, vascular ingrowth assay, and GSIS data were analyzed using the non-parametric Wilcoxon rank-sum test. Conditions in 2D and 3D GSIS were normalized to cell number, and PCL scaffold conditions were normalized to total protein. Values not within the 2σ interval were seen as outliers and removed prior to analysis. The results are presented as standard error of the mean (SEM). Sequencing was performed using two biological replicates; other experiments were repeated at least three times. Using IPA, the activity, enrichment, and statistical significance of canonical pathways in the dataset was calculated in two ways: (1) ratio of dataset molecule number mapped to pathway divided by total molecule number mapped to canonical pathway; and (2) right-tailed Fisher’s exact test to calculate the probability that the association between dataset genes and canonical pathway is explained by chance alone. Dataset molecules meeting the log fold change cut-off of <−0.5 or >0.5 and *p*-value < 0.05 were considered for the analysis. R version 4.0.0 with additional packages tidyverse v1.3, ggpubr v0.4, and ggrepel v0.8.2 (https://www.r-project.org) was used for data analysis and presentation.

Statistical significance is depicted with asterisks. The *p*-values are given as *p < 0.05, **p < 0.01, ***p < 0.001.

## Results

The experimental concept of this study was subdivided into hybrid scaffold fabrication, cellular integration, and functional evaluation of building-blocks for a proposed macrodevice. 3D-Printing was used for fabrication of an outer shell PCL component. The solid polymer component was surface-heparinized, leading to enhanced cell adhesion and hydrophilicity. 3D-Bioprinting was used to manufacture the inner, cell-encapsulating hydrogel structure of the hybrid scaffold (Supplementary Movie 1). Cellular integration of INS-1 cells in the hydrogel led to formation of proliferative pseudoislets. RNA sequencing revealed upregulation of proliferative and ß-cell-specific signaling, downregulation of apoptotic marker genes, but also transient metabolic stress post printing. Functional evaluation of the building-blocks was performed by GSIS and analysis of viability, function, and vascularization of xenografts *in ovo*. Hereby, the creation of a natural cellular niche by co-culture of INS-1 with endothelial cells (EC) resulted in enhanced insulin secretion upon glucose stimulation. Functional evaluation of the concept for future *in vivo* application was performed by computer simulation of Langerhans islets.

### 3D-Printing and heparin functionalization of PCL improved applicability

**Figure 1.**
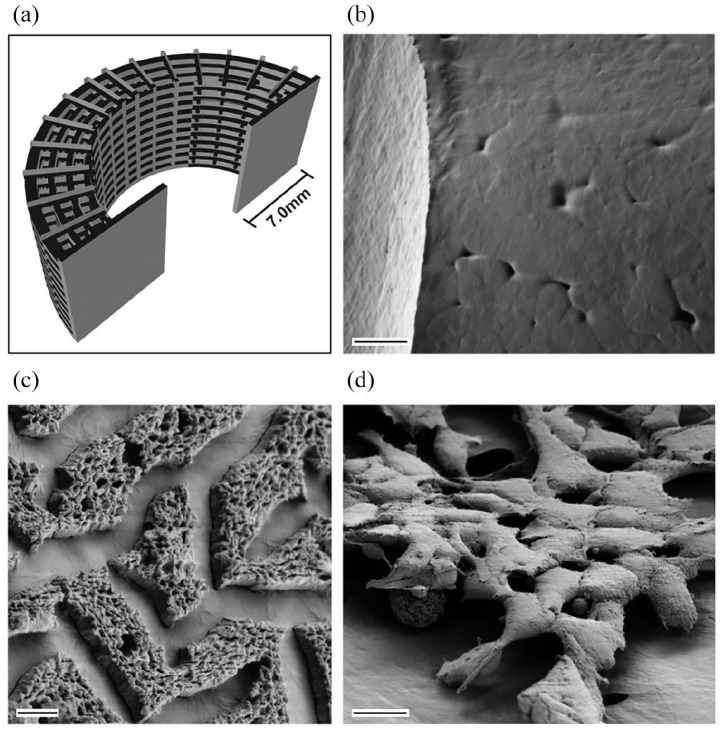
Solid polymer scaffold component was functionalized to enhance applicability, cell adhesion, and vascular ingrowth. (a) CAD model of PCL scaffold for 3D-printing as used in the experiments. (b) Scanning electron microscopy of untreated PCL scaffold structure after 3D-printing shows smooth surface with minimal porosity. (c) Surface modification of 3D-printed PCL scaffold with heparin. (d) Adhesion of INS-1 on 3D-printed PCL scaffold. Scale bar (b) 20 µm, (c) 2 µm, and (d) 10 µm.

The FDA-approved PCL was chosen as the solid polymer component, the outer shell, of the building-blocks, based on criteria as described previously such as biocompatibility, mechanical strength, retrievability, potential for modification and functionalization with supplementary substrates such as growth factors, and fabrication of a suitable scaffold architecture within a scalable process.^7,11^ 3D-Printing with additional sacrificial support material (polyvinyl alcohol) enabled fabrication of precise, complex geometries (Figure 1(a), Supplementary Figure 1A). Due to hydrophobic characteristics, further modification by heparin conjugation was performed prior to cell seeding. Covalent binding of heparin (Hep-PCL group, Figure 1(c)) is shown by scanning electron microscopy compared with untreated controls (Figure 1(b)). Functionalization remained stable over time (4 weeks, storage in PBS; not displayed). We investigated the ability of 3D-printed PCL to support cell adhesion and function in cell culture using INS-1 and HUVEC (INS-1 cell adhesion displayed in Figure 1(d)). INS-1 adhesion could be observed on untreated and heparinized scaffolds, whereas HUVEC adhesion was observed only in the Hep-PCL group (Supplementary Figure 1B). Functional analysis showed that INS-1 cells cultured on 3D-printed PCL remained glucose-responsive in heparinized and untreated controls at both basal and high glucose levels (Supplementary Figure 1C). Interestingly, increased insulin secretion after stimulation with glucose was observed in the Hep-PCL group (Supplementary Figure 1C).

### Bioprinting of insulin-secreting cells leads to pseudoislet morphology, viability, and growth of bioprinted insulin-secreting cells

**Figure 2.**
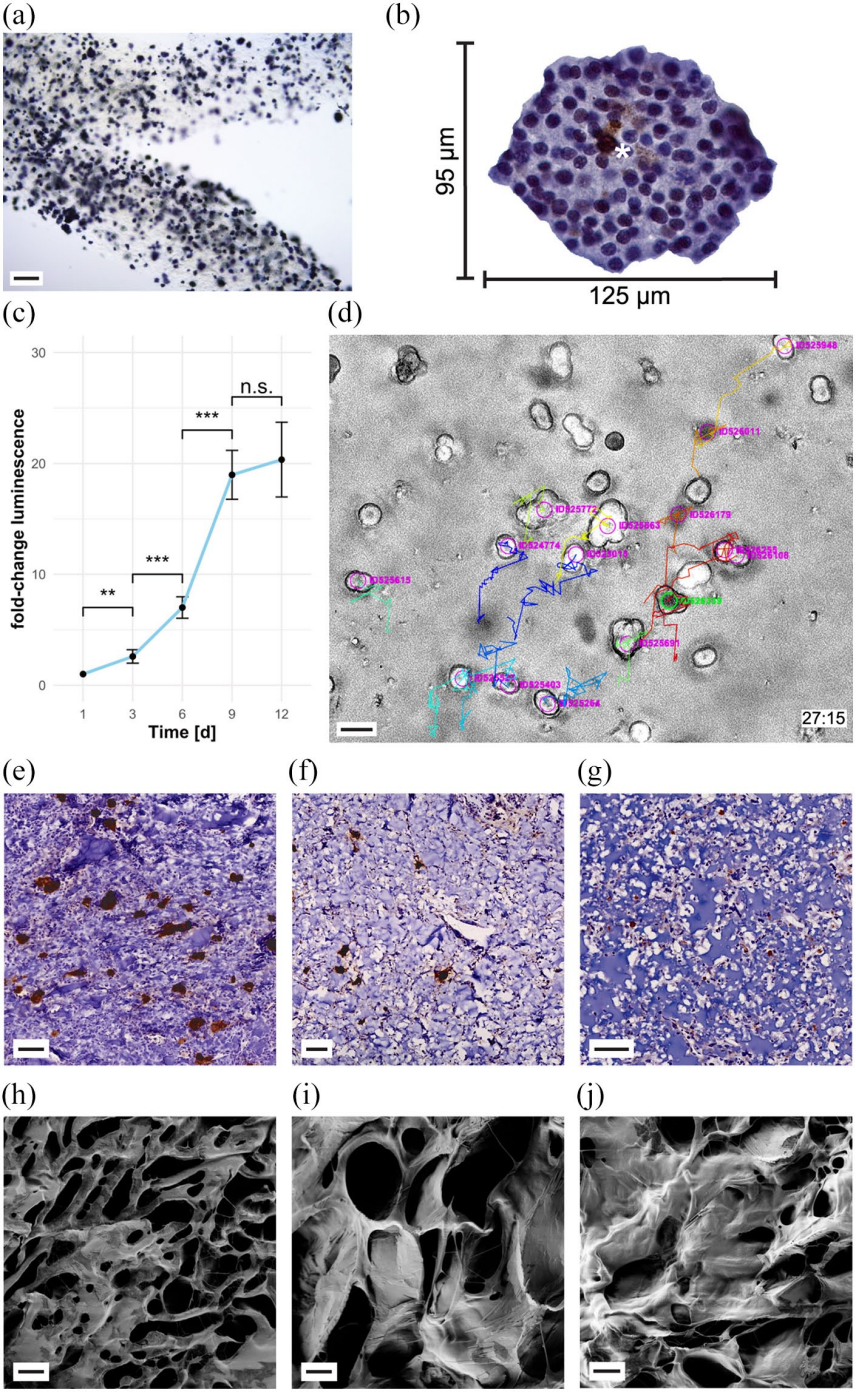
Hydrogel structure initiates pseudoislet formation. 3D-bioprinted INS-1 cells remain viable, migrate and proliferate. (a) MTT assay of pseudoislets formed after bioprinting (day 5 post-printing) of INS-1 containing gelatin methacrylate hydrogel shows metabolically active pseudoislets spatially distributed in 3D matrix. For further data s. Supplementary Figure 3 (b) Immunohistochemical staining of cleaved caspase-3 (brown, asterisk). Depicted: Pseudoislet formed from bioprinted INS-1 cells cultivated for 12 days. Immunohistochemistry revealed few apoptotic cells in the core of the pseudoislet. Apoptotic single cells disseminated in the hydrogel structure could not be detected. For further data s. Supplementary Figure 4 (c) Proliferation assay of bioprinted cells. A plateau is reached after 9 days in culture, indicating maximum loading capacity. Error bars depict SEM, *n* ⩾ 13/timepoint. (d) Cell movement tracking of time lapse microscopical investigation with TrackMate. Colored movement tracks (27:15h after start of experiment) depict, that cells encapsulated in hydrogel, bioprinted, and 2 s UV-cured (here: day 3 post-printing) are able to migrate within the hydrogel. Live microscopy was performed at 328 time points with an interval of 5 min. For further data s. Supplementary Movie 2. (e–g) Immunohistochemical anti-insulin staining (brown areas) of CAM assay explants of bioprinted INS-containing hydrogel (e) after 2 s of UV curing, (f) 5 s of UV curing, and (g) 15 s of UV curing. In INS-containing hydrogel with 2 s crosslinking time (e) most insulin-stained areas were detected and pseudoislet formation is found. Spatially distributed pseudoislets remained viable and functional. Longer crosslinking periods (5 s (f), 15 s (g) showed few pseudoislets (f) or scattered INS-1 single cells (g). (h–j) Scanning electron microscopy of freeze-dried hydrogel structures (GelXA LAMININK 411) with different UV-curing times. Electron microscopy showed different morphological features of the hydrogel depending on the crosslinking time of (h) 2 s, (i) 5 s, (j) 10 s. The hydrogel micro-structure showed a homogenous porous structure (h). For further data s. Supplementary Figure 5A–C. Scale bar (a) 200 µm, (d) 20 µm, (e–g) 50 µm, and (h–j) 10 µm.

In addition to a solid polymer component, the inner core of each building-block contains a cell-encapsulating hydrogel. 3D-Bioprinting of INS-1 cells in gelatin methacrylate blended with laminin-411 and subsequent UV crosslinking (405 nm) resulted in a 3D-architecture encapsulating spatially distributed single cells (Supplementary Movie 1 and Figure 2). After cultivation for 5 days, insulin-secreting cells in the bioprinted hydrogel started to form INS-1 cell-clusters (following named pseudoislets) (Figure 2(a), Supplementary Figure 2). These multicellular aggregates grew up to, but did not exceed, the size of average islets of Langerhans (Figure 2(b), islet equivalency commonly stated as 150 µm, Supplementary Figure 3).^4,27,28^ A diminishing number of remaining single cells permits the conclusion that cells are able to migrate within the hydrogel (Supplementary Figure 2). Live microscopy imaging of cultivated cells encapsulated in hydrogel confirmed proliferation and migration of cells after bioprinting (Figure 2(c), Supplementary Movie 2). After long culture periods (>10 days) INS-1 pseudoislets could be observed to migrate out of the hydrogel structure due to the extensive pseudoislet density. For qualitative visualization of cell viability after printing and metabolic activity in 3D hydrogel culture, an MTT assay was used. Figure 2(a) shows a sector of a 3D-bioprinted hydrogel grid with encapsulated, metabolically active pseudoislets (MTT results of day 7 post-printing in Supplementary Figure 3). Unstained pseudoislets or single cells were not detected. Quantitatively, the viability and proliferation of pseudoislet structures were measured with cytoplasmic ATP-based CellTiter-Glo® 3D Viability Assay. Consistent with morphological observations, a significant 19-fold increase of luminescent signal was observed in the first 9 days and reached a plateau on day 12 (Figure 2(c)). Proliferation of cells relies on sufficient oxygen and nutrient diffusion into the hydrogel structure to allow an entry into the cell cycle.^29^ Once maximum cellular density has been reached, pseudoislets start to migrate out of the hydrogel. Precise investigation of apoptosis of encapsulated cells after bioprinting by cleaved caspase 3 staining confirmed the absence of apoptotic single cells scattered in the hydrogel (Supplementary Figure 4). At a plateau of pseudoislet growth 12 days post-printing, scattered cells within the pseudoislets were positive for cleaved caspase 3 indicating at least a pre-apoptotic state (Figure 2(b), Supplementary Figure 4). Stained cells were merely located in the core of pseudoislets. Viability and proliferation of cells depends on, among other factors, the biomechanical properties such as porosity of the encapsulating material. In order to tune the UV-curing based 3D-bioprinting process, the effect of different crosslinking times (2–15 s) on cell behavior and material properties were investigated. Immunohistochemical stainings were applied to study the morphology of cell-containing hydrogels (Figure 2(e)–(g)), while the microstructure and diffusion capacity of the gels were investigated using scanning electron microscopy (Figure 2(h)–(j), Supplementary Figure 5 A-C) as well as an diffusion assay (Supplementary Figure 6). As expected, samples exposed to different UV-light crosslinking times exhibited differences in the respective features. Although it might appear obvious, UV curing itself, at least in the doses used here, led to no significant differences in terms of cell survival (2D monolayer culture of INS-1 cells at different crosslinking times compared to negative control; trypan blue cell counting; not displayed). Still, with respect to cell proliferation and pseudoislet formation qualitatively best results were achieved using the lowest (2 s) crosslinking time (Figure 2(e)). Even though a crosslinking time of 5 s per layer resulted in a hydrogel with viable pseudoislets (Figure 2(f)), the number of islets is reduced compared with lower UV-light exposure times (e.g. 2 s). Even higher UV-light doses (15 s exposure time) resulted in a gel structure appearing dense with no pseudoislet formation and a reduced number of INS-1 cells at the end of the experiment (Figure 2(g)). The described findings are in line with the results of the samples’ microstructure as well as their diffusion capacity. All samples exhibit a highly porous microstructure with intra-sample homogenous sponge-like networks. However, qualitative inter-sample comparison suggests a crosslinking time dependent variation of the sample porosity. The described findings were further supported by the conducted diffusion assay. With increasing UV-light exposure the diffusion capacity was significantly reduced from 650 µm (2 s) to 240 µm (10 s). The results provide further evidence for the strong influence of the crosslinking time on the material’s porosity.

### Transcriptome analysis of 3D-bioprinted insulin-secreting cells reveals enrichment of proliferative, anti-apoptotic pathways, structural integrity, and ß-cell function

**Figure 3.**
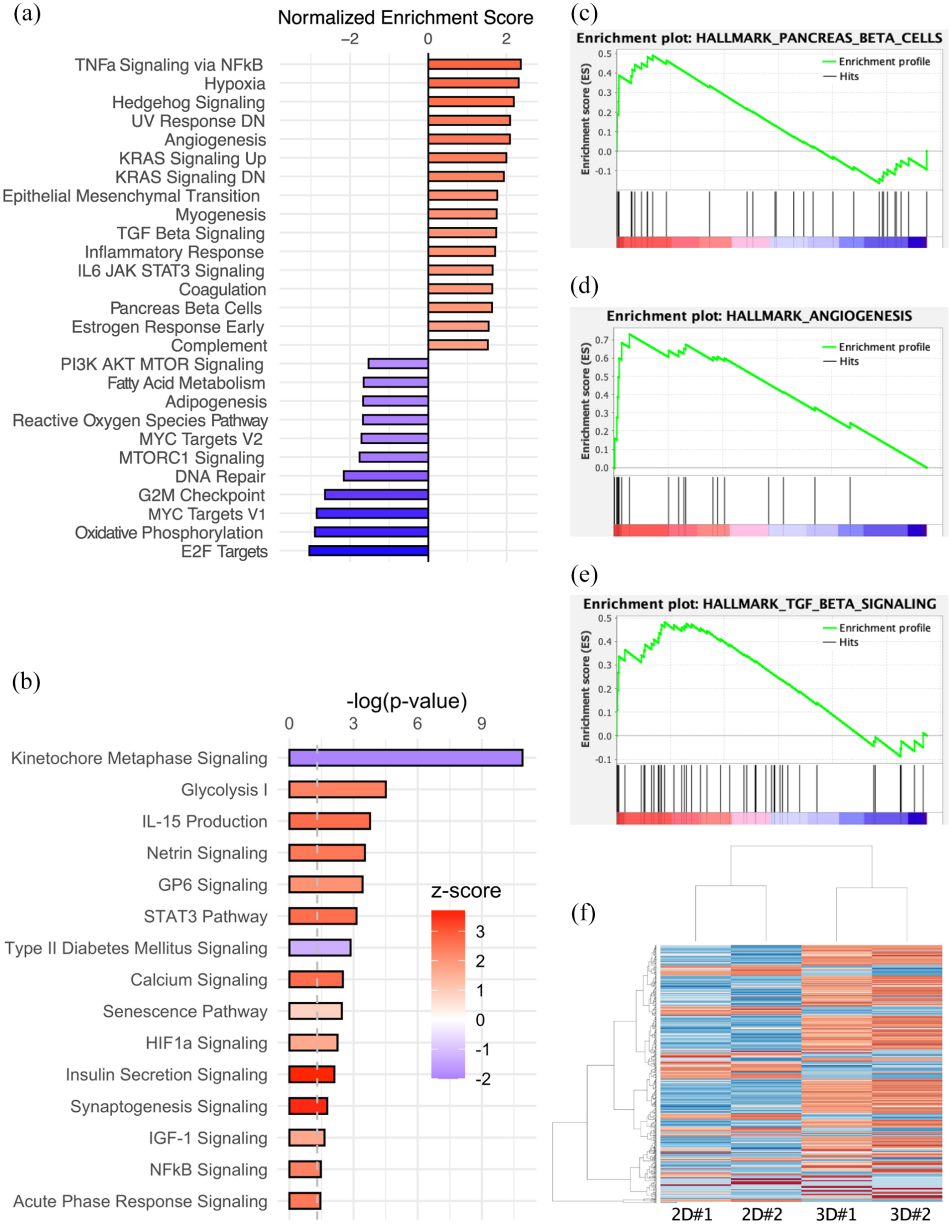
mRNA sequencing of 3D-bioprinted domes compared with monolayer culture control shows robust differential gene expression clusters and alteration of hallmark pathways. (a) Gene set enrichment analysis revealed significantly altered hallmark pathways (*p* < 0.05). (b) IPA revealed significantly altered canonical pathways (*p* < 0.05) and color-coded *z*-score indicates activation of pathways including insulin secretion signaling. Upregulated hallmark pathways include pancreas ß-cells (c), TGFß signaling (d), and angiogenesis (e). (f) Heatmap of alteration in gene enrichment (500 most significant alterations) comparing 3D-bioprinted culture with monolayer culture.

For a comprehensive understanding of the structural and functional implications of 3D-bioprinted hydrogel building-blocks including insulin-secreting cells, global gene expression analysis (total mRNA) was performed using next-generation sequencing. 3D-Bioprinted grid structures with integrated insulin-secreting cells (Supplementary Figure 7A) were compared with their counterparts grown in monolayer (Figure 3(a) and (b)). Differential gene expression analysis and subsequent gene enrichment analysis revealed significant alterations of predefined hallmark pathways (Figure 3(a), c–f; for MA-plot, see Supplementary Figure 7B).^30^ Sixteen hallmark pathways, including the sets for “TNF alpha signaling via NFkB,” “hypoxia,” “TGFß signaling,” and “pancreas ß-cell,” were significantly upregulated, whereas 11 pathways, including sets for “adipogenesis,” “reactive oxygen species pathway,” and “oxidative phosphorylation,” were downregulated in 3D-bioprinted cells cultivated in hydrogel compared to monolayer control (Figure 3(a); full report in Supplementary Figure 7C). The results are quantified using normalized enrichment score (NES)- and false detection rate (FDR)-adjusted p-values with a cut-off of 5% for hallmark gene sets. Ingenuity Pathway Analysis (IPA) revealed 100 significantly affected canonical pathways (cut-off threshold *p* < 0.05) (Figure 3(b); full report in Supplementary Figure 7D). A graphical network summary of the major biological themes for exploratory purposes is displayed in Supplementary Figure 7E. Regarding the effects of 3D-bioprinted hydrogel culture and the accompanying microenvironment that led to pseudoislet formation, evaluation of the enrichment of the pancreas ß-cell-specific gene set (NES 1.63, *p* < 0.01) is of special interest (Figure 3(c)). An upregulation of processes including regulation of insulin gene expression, glucose transport and sensing, modulation of ATP-sensitive potassium channels, secretory processes, and ß-cell-specific canonical transcription factors and promotors was found (e.g. *Slc2a2*, *Sur1*, *Kcnj11*), whereas no significant difference in insulin gene expression itself could be detected (see excerpt table of differential gene expression in Supplementary Figure 7G). ß-Cell-specific pathways were unbundled using IPA with Ingenuity Knowledge Base as reference and revealed an activation of insulin secretion signaling (*z*-score 3.657, *p* < 0.01) and IGF-1 signaling (*z*-score 1.667, *p* < 0.05) and an inactivation of type 2 DM signaling (*z*-score −1.265, *p* < 0.01). In contrast to *Mafa* and *Neurod1*, *Pdx1* is downregulated compared with monolayer control. However, causal network analysis identified all three transcription factors to be activated master regulators (activation *z*-score 3.182, bias-corrected *p* < 0.0001) based on 32 downstream genes in the dataset. The significant overexpression of IGF/TGFß signaling cascade-related genes puts *Pdx1* expression in relation. IGF/TGFß signaling is an important proliferative and functional regulator of ß-cell growth and proliferation, and the enrichment data correlate with our findings in growth assays (TGFß signaling NES 1.74, *p* < 0.01; Figure 3(e)).^31^ Prolonged hyperglycemia induces glucotoxicity.^32^ An accumulation of glucose in 3D hydrogel culture may have led to GSK3B-regulated PDX1 phosphorylation and thus faster proteasomal degradation. High glucose induces FOXO1, which has been described to integrate ß-cell proliferation with adaptive function to maintain tissue homeostasis at the center of IGF/TGFß signaling.^31,33^ Prolonged hyperglycemia may have triggered a transient inflammation of insulin-secreting cells, in which TGFß1 interacts in the NFkB pathway (TNFA signaling via NFkB NES 2.37, *p* < 0.01).^31^ Additionally, overexpression of *Irs2* and *Atf3* has previously been reported to have a protective effect on ß-cells under metabolic demand and alleviate hyperglycemia-induced apoptosis in support of our hypothesis.^34^ We hypothesize that INS-1 cells exposed to environmental stress due to the bioprinting process, transient hypoxic conditions, and hyperglycemia may have activated HIF1a/PFKFB3 stress-repair signaling.^35,36^ This transient state is an adaptive, protective metabolic response that slows ß-cell death at the expense of ß-cell function and activates glycolysis (*z*-score 2.333, *p* < 0.0001) while inhibiting the oxidative phosphorylation (NES −2.90, *p* < 0.01).^35,36^ Additional upstream regulator analysis supported the likelihood of our hypothesis identifying glucose (activation *z*-score 3.835, overlap *p* < 0.0001) based on expression of 87 genes in the dataset (see the graphical display of glucose upstream regulator network incl. exemplary pathway overlay in Supplementary Figure 7F). Further, pseudoislet formation in 3D-bioprinted hydrogel culture led to a significant decline of pro-apoptotic genes *Bax*, *Bad*, and caspase 3 and overall activation of the functional annotation “Cell Viability” (*z*-score 2.160, *p* < 0.0001) in IPA. This finding is consistent with cleaved caspase 3 staining showing only scattered apoptotic cells in the core of pseudoislets. Pseudoislet formation and proliferation in culture, together with reduced pro-apoptotic gene expression, may be a result of reduced anoikis due to cell-cell and cell-matrix interactions and anchorage-dependent growth. In islets, laminin-411 and laminin-511 are expressed and have been suggested to play an important role in β-cell proliferation and insulin transcription.^37,38^ A hydrogel blend containing laminin-411 was therefore used in this study. The transcriptome data revealed significant overexpression of fibronectin, E-cadherin, basal cell adhesion molecules, and laminins secreted by INS-1 and known to be essential for structural integrity and cell contacts of islets, thereby enhancing functionality.^37–39^ In addition, significant overexpression of extracellular matrix (ECM)-localized growth factors such as *Vegfa* was found in INS-1 cells in 3D culture.

### Xenotransplantation to fertilized chicken eggs results in extensive vascular ingrowth and neoangiogenesis in scaffold components

**Figure 4.**
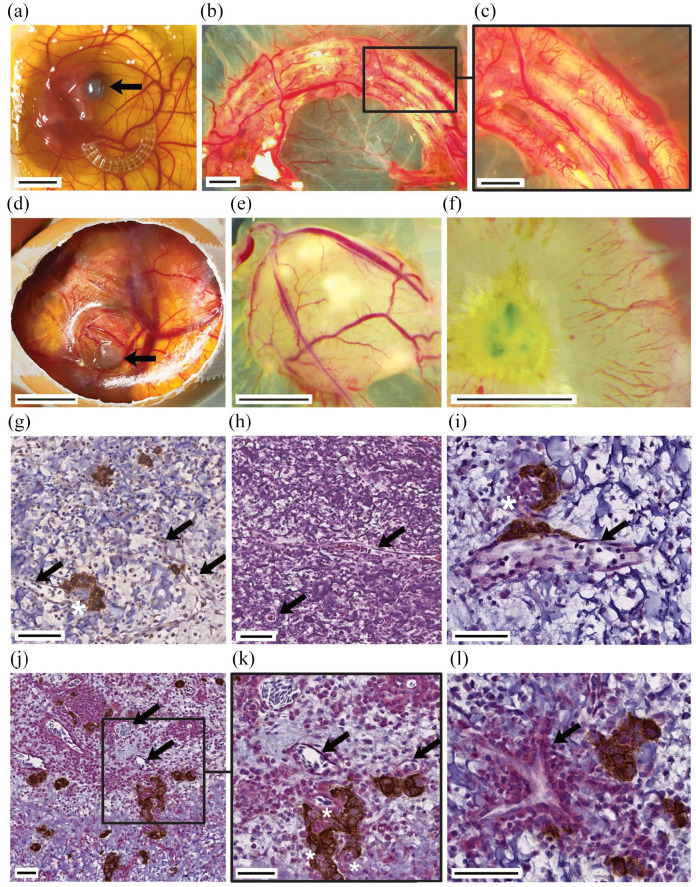
Chorioallantoic membrane assay is a suitable model for investigating angiogenesis in tissue-engineered grafts. Extensive, rapid vascular ingrowth is seen in both PCL and cell-laden hydrogel structure after the 9-day assay period. *Ex ovo* CAM assay experiments enabled direct comparison of heparinized PCL scaffolds with untreated controls and validated the beneficial properties of heparinization for enhanced vascular ingrowth ((a–c) arrow indicates eye of chicken embryo). *In ovo* CAM assay experiments were used for investigation of 3D-bioprinted INS-1-laden droplets ((d–l) arrow indicates xenotransplant). Vascular structures (arrows) penetrated into the scaffold (g–l). (g) Anti-insulin immunohistochemical staining of CAM assay explant. (h) H&E staining of CAM assay explant. (i–l) Anti-insulin (brown) and anti-CD31 (red) immunohistochemical double-staining of CAM assay explant. Rapid vascularization maintained viability and function of pseudoislets. Peri- and intra-insular (asterisks) vessels were detected (g–l). Scale bar (a and d) 10 mm, (b, c, e, and f) 2 mm, (j) 100 µm, (g, h, k, and l) 50 µm, (i) 20 µm.

Vascularization of building-blocks is crucial for the viability and function of islets.^5,9,10,40^ The transplantation of the PCL scaffold (Figure 4(a)–(c)) and hydrogel (Figure 4(d)–(f)) to the CAM of fertilized chicken eggs was utilized to investigate vascular ingrowth and vessel penetration through the solid PCL channel architecture *in vivo* (Figure 4, Supplementary Figure 8A). The surface-functionalized PCL had beneficial effects on vascularization, as shown by the significantly enhanced total vessel network length (untreated control: 26,856 ± 3502SD [px], Hep-PCL: 34,766 ± 1650SD [px], *p* < 0.05) (Supplementary Movie 3 and Figure 8A). Especially in the initial period after implantation, cells are faced with hypoxia and diffusion-based supply.^41^ We showed that insulin-secreting pseudoislets cultured in hydrogel structure survived this initial period before formation of vascularization. After 9 days of *in ovo* cultivation (Supplementary Figure 8B), the xenografts were explanted. A dense host-derived vascular network surrounding the xenograft was observed (Figure 4(e)). Stereomicroscopy showed that some vessels penetrated the xenograft (Figure 4(f)–(l)). Subsequent immunohistochemical analysis proved not only extensive peri-islet vascularization but also intra-islet vascularization (Figure 4(g), (j), and (k)). The close spatial relationship of insulin-positive pseudoislet structures with newly formed vascular structures (CD31+) is displayed in double stained xenotransplant specimens (Figure 4(i)–(l)). Additionally, staining with avian anti-CD34 showed capillary sprouting at the periphery and migration of host EC toward the graft center without additional external mitogenic stimulation (Supplementary Figure 8C). A direct comparison of heparin-conjugated and control scaffolds was possible in *ex ovo* CAM assay, where an anticoagulation effect of the heparinized scaffolds was observed (Supplementary Figure 8B, EDD11/18).

### Survival, viability, and insulin secretion of pseudoislets *in ovo*

3D-Bioprinted INS-1 cells formed pseudoislets *in vitro*. Pseudoislet formation was also observed after excision of *in ovo* cultured bioprinted hydrogel droplets. Xenografts remained on the CAM for 9 days supplied with nutrients only by diffusional processes from the CAM and newly formed vascular structures penetrating the xenograft. The CAM assay modeled the initial period after implantation, in which comprehensive vascularization of the graft is yet to develop. Insulin-secreting pseudoislets survived this initial period. Immunohistochemical staining against insulin demonstrated that pseudoislets remained functional until explantation (Figures 2(e) and 4(g), i-l). However, only pseudoislets with either minimal diffusion distance to the surrounding tissue (max. 650 µm; Supplementary Figure 9) or contact to intra-graft vessels (Figure 4(i)–(l)) were found, therewith decreasing the homogeneity of the cellular distribution. Pseudoislets located deep inside the hydrogel structure experienced a hypoxic environment consistent with previous literature findings.^41^ Over a period of 9 days *in ovo*, anaerobic respiration was presumably insufficient to meet metabolic demand. Dependence on diffusion of nutrients and gases, soluble factors, and waste products from surrounding tissue into the graft limits the size if cell viability is to be retained.^29,41^ Homogeneous spatial distribution of pseudoislets in cross-sections of the graft was observed both at 20 µm and 40 µm distance from the bottom (Supplementary Figure 9). The graft area in these cross-sections of about 1.3 mm^2^ and 1.8 mm^2^, respectively, and the relatively small diffusion distance from graft base to CAM resulted in sufficient viability. At 60 µm diffusion distance, pseudoislets were predominantly located in marginal areas of the graft cross-section (6.45 mm^2^; Supplementary Figure 9). In addition, it is notable that pseudoislets are reduced in size with increasing diffusion distance. Pixel classification segmentation detected 1.8%, 2.1%, and 2.1% of insulin^ +^ area at 20, 40,and 60 µm cross-section layers, respectively. Examination of explanted specimens in which either the bioprinted graft had been UV-cured for a longer period (15 s) or a larger graft structure had been printed confirms the findings stated above (Supplementary Figure 9).

### Co-culture with EC ameliorates insulin secretion

**Figure 5.**
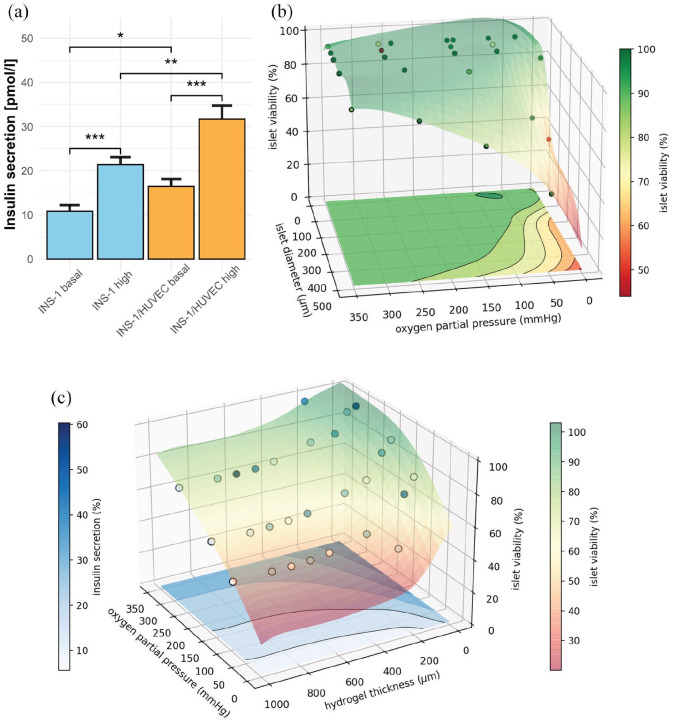
Insulin-secretion of bioprinted INS-1 cells is enhanced in co-culture with EC. Computer simulation of human Langerhans islets predicts concept feasibility and defines boundary conditions for viability and function of bioprinted 3D hydrogel geometries. (a) Insulin secretion of hydrogel-embedded INS-1 culture and INS-1/HUVEC co-culture at basal and high glucose levels 3 days post printing. 3D-Bioprinted, encapsulated INS-1 cells are responsive to glucose stimulation. The INS-1/HUVEC co-culture ameliorates the amount of insulin secreted. Insulin levels for both experimental settings were normalized to 10,000 cells. Error bars depict SEM, *n* ⩾ 20/condition. (b) Simulation of viability of human islets of Langerhans encapsuled in hydrogel by finite element analysis. Calculation for 400 µm constant diffusion distance through the hydrogel. Glucose inflow concentration is kept at 10 mM. Inflow oxygen partial pressure ranging from 5 mmHg to 350 mmHg, islet diameter from 100 µm to 500 µm. Circles represent data points, gathered by diffusion simulation, and colored semitransparent surface and projection on the base represent fit through cell viability data. (c) Simulation of insulin secretion and viability. Constant glucose concentration of 10 mM, constant islet diameter of 500 µm, inflow oxygen partial pressure varying between 50 mmHg and 350 mmHg, hydrogel shell thickness ranging from 0 µm to 1000 µm. Circles represent calculated datapoints, semitransparent surface represents fit through 3D cell viability (right color bar). Insulin secretion is displayed as contour lines at the bottom of the diagram (left color bar). Simulations were performed for 464 different scenarios.

The effect of the extracellular microenvironment and cellular crosstalk on the functional outcome was investigated by means of GSIS (Supplementary Figure 10A). The 3D-bioprinted INS-1 group responded to glucose stimulation by secretion of 10.8 and 21.4 pmol/l insulin in basal and high glucose concentration, respectively (*p* < 0.001, Figure 5(a)). The functional outcome shows that cell encapsulation, bioprinting, and UV-crosslinking did not prevent the insulin-secretory function of bioink droplets (INS-1 group). The 3D-bioprinted co-culture group with HUVEC (INS-1/HUVEC, 1:2 ratio) yielded a secretion of 16.4 pmol/l and 31.7 pmol/l (*p* < 0.001, Figure 5(a)). At both basal and high glucose concentrations, the co-culture group showed significantly enhanced secretion of insulin compared to INS-1 group (*p* < 0.05, *p* < 0.01). Both groups showed significantly increased insulin secretion after high-glucose treatment compared with basal glucose treatment (*p* < 0.001). Interestingly, the 2D-monolayer culture showed higher absolute insulin secretion after glucose challenge, possibly due to the tumor origin of the 2D adhesive cell line, a higher surface to volume ratio, and enhanced stimulus conduction of confluent monolayer cells (Supplementary Figure 10B). However, the stimulation indices (high glucose/basal glucose ratio) were increased in both bioprinted 3D culture conditions (1.56 [2D INS-1] compared with 1.97 [INS-1] and 1.93 [INS-1/HUVEC]). The stimulation in both 3D culture groups is similar to the characteristic index of healthy and freshly isolated human islets and can be attributed to the 3D matrix microenvironment.^42^

### Computer-aided applicability screening of scaffold architecture by finite element analysis demonstrates feasibility for Langerhans islets

The use of INS-1 cells instead of human islets could be viewed as a shortcoming of this study. Especially in terms of oxygen and nutrient diffusion the variable size of human islets^4^ needs to be considered to avoid function loss and central core necrosis.^3,43^ We addressed this requirement by performing simulations of diffusion gradients and metabolic activity of human islets. The intention was to enable different boundary conditions for variations of parametric scaffold architecture applicability, thus avoiding iterative experimental designs of a macroscopic device from bench to bedside. A primary limitation in translation of all tissue engineering approaches, in particular macrodevice scaffolds, is vascularization.^4^ According to previous findings and results presented here, the diameter of the hydrogel capsule must extend no more than a few 100 µm for sufficient oxygen diffusion maintaining pseudoislet viability.^44,45^ The applicability of the scaffold architecture was validated by *in silico* modeling based on literature data^24^ (parameters displayed in Supplementary Figure 11). Finite element simulations with multiple input parameters predicted viability and insulin secretion of bioprinted islets of Langerhans as function of oxygen partial pressure, surrounding hydrogel thickness, and the diameter of the islet itself for 464 scenarios. Our simulations focused on the critical initial period after implantation, in which vascular ingrowth has not yet occurred. Hence, only diffusion processes were considered. Hypoxic conditions result in decreased viability and function, especially for large islet structures (Figures 1, 5(b) and (c)). Oxygen and glucose diffusion through islets is hampered compared with hydrogel diffusion (Supplementary Figure 11). Thus, large islets are more severely affected by hypoxia. This is consistent with our findings, that showed few apoptotic cells to be located in the core of large pseudoislets *in vitro*. As concluded before,^24,27^ small to average-sized islets containing bioink xenografts are more likely to survive. To a lesser yet considerable degree, cell viability with respect to oxygen levels is reduced with increasing hydrogel diffusion distance (Figure 5(c), Table 1). Finite element simulation then enabled identifying the best fit for bioprinting geometries. Insulin secretion by islets decreases rapidly with declining oxygen partial pressure and is diminished before viability begins to decrease. With a more than four times smaller diffusion coefficient, glucose diffusion is a more limiting factor for insulin secretion. However, simulations with hyperglycemic glucose concentrations showed decreased viability and function of islets (Table 1). High glucose concentrations stimulate insulin secretion in islet ß-cells, leading to increased oxygen consumption. ß-Cells in the islet shell consume more oxygen in response to hyperglycemia, leaving a smaller amount for core cells. Thus, islet core oxygen levels fall below a critical boundary for cell viability and cause core necrosis. The simulated functional outcome is additionally dependent on hydrogel thickness for insulin outflow. It is important to consider that due to the larger size of insulin, the overall secretion from the xenograft is also limited by accumulation and degradation of the hormone in the bioprinted hydrogel. The pseudoislets generated in this study and islet-like organoids generated from pluripotent stem cells^46^ range up to 150 µm in diameter and thus present more suitable prerequisites for diffusional nutrient supply while encapsulated.^28^

**Table 1.** Finite element analysis to prove feasibility for human Langerhans islets (excerpt).

Oxygen level (mmHg)	Glucose concentration (initial) (mM)	Hydrogel thickness (µm)	Islet diameter (µm)	Viability (%)	Insulin secretion (%)
160	10	50	500	92.1	46.53
160	10	50	150	100	70.24
90	10	50	500	78.5	32.46
90	10	50	150	100	70.24
90	5	50	500	87.2	14.10
90	25	50	500	73.8	41.26
90	5	500	500	54	3.82
90	25	500	500	40.3	8.25
90	5	1000	500	51.2	3.12
90	25	1000	500	37.5	6.2
90	5	50	150	100	28.76
90	25	50	150	100	95.96
90	5	500	150	100	27.61
90	25	500	150	100	69.56
90	5	1000	150	100	27.6
90	25	1000	150	99.8	64.85

Anticipated results for cell viability and insulin secretion as function of oxygen level, surrounding glucose concentration, thickness of hydrogel encapsulation, and islet diameter.

## Discussion

**Figure 6.**
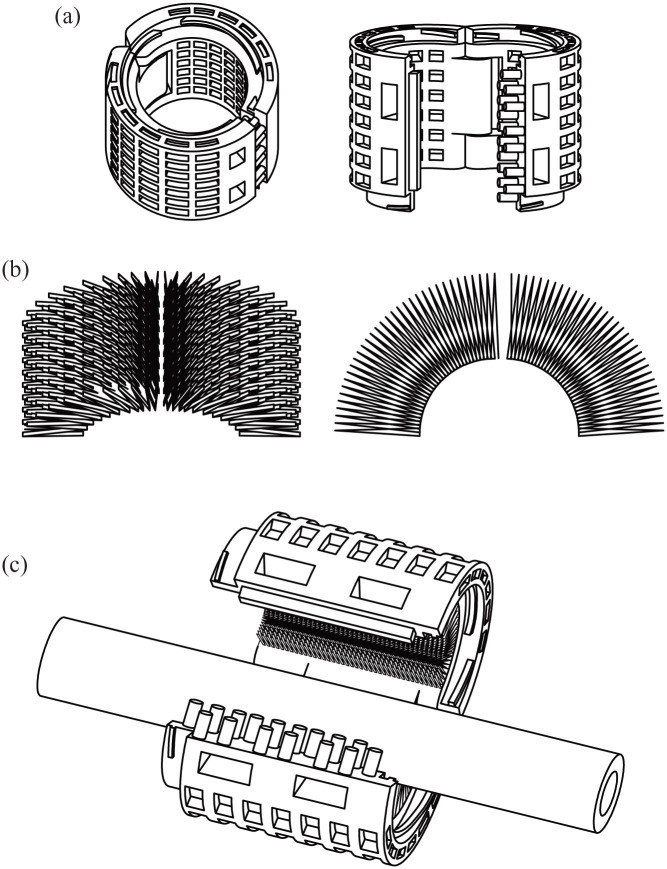
Proposed application concept of a perivascular implant based on a hybrid scaffold building-blocks. (a) CAD-drawing of polycaprolactone shell component: Proposed 3D-printed outer layer. (b) CAD-drawing of cell-encapsulating hydrogel structure: Proposed bioprinted inner layer. (c) CAD-drawing of proposed merged hybrid perivascular device for future application in human. In this study, building-blocks for a future insulin-producing perivascular implant were investigated *in vitro* and *in ovo*. In further studies *in vivo*, we propose merging the building-blocks to form a hybrid implant as depicted here. Such an implant could be able to encase neuro-vascular structures. The modular design, as proposed in the CAD-drawing could easily be implanted to encase bodily structures and would allow further hybrid devices to be attached along the bodily lead structure and thus being a building-block for a patient-specific implant itself. For further information on the proposed concept see Supplementary Movie 4.

In the present study, we developed and evaluated a concept for 3D-(bio)printing of insulin-secreting tissue as a novel treatment option for patients with insufficient insulin-secretory function. We proved the feasibility of our building-block concept with regard to hybrid scaffold fabrication, integration of cells, and functional evaluation *in silico*, *in vitro*, and *in vivo*. Hybrid scaffold building-blocks were fabricated by 3D-(bio)printing using PCL and gelatin methacrylate hydrogel. In this concept, we propose using a permeable outer shell of a PCL mesh (Figures 1(a) and 6(a)) to support the inner insulin-secreting hydrogel core (Figures 2(a) and 6(b)) until it can support itself (for proposed future application concept see Supplementary Movie 4). Solid PCL as retrievable outer shell is a logistic template for a hybrid macrodevice. PCL is FDA-approved as a drug delivery device and is used in medical devices because of its biocompatibility, low immunogenicity, low foreign body response, and physicochemical properties.^7,11^ Covalent heparin binding on the PCL surface improved vascularization *in vivo* and, in agreement with a previous study by Marchioli et al.,^7^ increased cell adhesion due to decreased hydrophobic properties, increased protein binding capacity, and different surface topography. EC coating of polymers has been reported to accelerate vascularization.^6^

Cellular integration was investigated by means of morphology, viability, proliferation, migration of cells, apoptosis staining, and transcriptome alterations. The bioprinted, multicellular hydrogel provides a microenvironment for insulin-secreting cells to form islet-like structures, thrive, and function. Total mRNA sequencing revealed that morphological pseudoislet formation in 3D culture correlates to upregulation of ß-cell-specific proliferative pathways, insulin secretion signaling cascades, and angiogenesis pathways. The complex regulation and interaction of ß-cell-enriched genes impedes a functional assessment of 3D-bioprinted hydrogel culture by transcriptome analysis alone. ß-cell dedifferentiation after prolonged hyperglycemic conditions is a key mechanism causing a loss of functional ß-cell mass in type 2 DM to alleviate apoptosis rates.^32^ However, we did not detect a functional consequence; dedifferentiation seems to be absent due to the overexpression of genes specific for the ß-cell phenotype and robust activation of ß-cell-specific pathways. This interpretation correlates with the findings from growth assays, apoptosis assays and stimulation index in GSIS experiments. Instead, the pseudoislets in 3D culture seem to respond to the metabolic demand by compensatory upregulation of *Foxo1*, *Iapp*, *Irs2*, and *Atf3* expression. VEGF-A, synthesized by ß-cells among pancreatic cell types, is a crucial factor for communication with EC, and VEGF-A-depleted islets are only inefficiently vascularized when transplanted into a host compared with wildtype control.^37,40^ In addition, islets depleted of *Vegfa* have a reduced number of capillaries and exhibit several defects in β-cell function, including insulin transcription, insulin content, first-phase insulin secretion, glucose tolerance, and β-cell proliferation.^37,40^ In EC, VEGF-A will induce cell migration, proliferation, maintenance of capillary fenestrations, and ß-cell mass.^3,5,37^ During clinical islet isolation the dense capillary network becomes disrupted.^5,28^ The capillary network or EC networks play an essential role in vascularization and thus survival of islets after transplantation.^5–7,28^ However, previous studies have shown a concentration-dependent adverse effect of additional artificial VEGF-A delivery.^7^ Our results show significantly upregulated expression of VEGF-A after cell integration in the hydrogel structure and extensive vascular penetration and neoangiogenesis *in ovo* (Figure 4(d)–(l)). The microenvironmental gradient of VEGF-A may already have reached a therapeutic gradient without external VEGF-A delivery. Our findings support omitting obligatory additional VEGF-A delivery in building-blocks, as overdosage will result in leaky and dysfunctional vessels.^5,9^

Functional evaluation of insulin secretion after glucose challenge showed the superiority of co-culture with EC in providing a natural cellular niche. The beneficial effect of EC on secretory function of insulin-secreting cells has been reported before.^5,37,38^ As an essential element of pancreatic vessels, EC contribute to the delivery of glucose as the primary input of ß-cell signaling and transport insulin as functional output to its target location. EC embedding in tissue engineered constructs has been reported to be beneficial for rapid neovascularization *in vivo*.^47^ In addition, direct and paracrine communication between ß-cells and EC enhances the structural and functional integrity of islets and their insulin-secretory function.^5,38^ Thrombospondin-1, endothelin- 1, and hepatocyte growth factor secreted by EC may have directly stimulated insulin secretion.^5^ In addition, ECM secretion by HUVEC influences the ß-cell microenvironment.^5,38^ As reported previously, external delivery of integral basement membrane proteins can compensate only partially for ECM and EC loss during islet isolation,^37^ and this explains the superior functional outcome of co-culture hydrogel compared to laminin-containing hydrogel with INS-1 alone. Due to the absence of an immune system in the developing chick embryo,^48^ CAM xenografts are not suited to assess interactions of the scaffold with the host immune system, including protection of insulin-secreting pseudoislets by hydrogel encapsulation itself. In the developmental stage used in the CAM assay, the chicken immune system is still developing and is not yet fully functional. Further investigations including *in vivo* trials are necessary. The feasibility of the concept and the building-block architecture for integration of human islets was evaluated by computer simulations using finite element analysis. *In silico* analysis of 464 different scenarios generated the boundary conditions for a geometry that is proposed for future perivascular *in vivo* application. Especially small and average-sized islets, similar to the pseudoislets formed by INS-1 cells, are feasible for integration into a bioprinting process, as hypoxic conditions in the core of larger islets reduce overall viability and function. Future experiments with human islets will be necessary to verify our findings with special regard to islet core necrosis. Scaffold devices have often been implanted in the subcutaneous space, resulting in low potential for vascularization and lack of glycemic control.^8,9,43^ In the overall concept, findings from this study are merged with findings from previously published evidence gap maps, showing that transplantation of scaffold-based constructs alongside or in close proximity to vascular structures has been rarely investigated.^3,8,43^ Although type 1 and type 3c DM cause a loss of insulin-secreting cells, the cell sources used for transplantation can differ.^2^ Transplantation of autologous islets in case of type 3c DM diminishes immunological reactions toward the graft.^2^ In the case of allogenic islets necessary to treat type 1 diabetes or other cell sources, however, more research on the immunoprotective capabilities of the device is required. If necessary, local immune modulation by functionalization of immunosuppressive agents as described earlier might be harnessed.^3^ Differentiation and integration of progenitor and stem cells as an unlimited cell source has the potential to expand the applicability of our concept and future bioconvergence research.^2,3^ Integration of cells into the hydrogel structure initiates pseudoislet formation at the microscopic level and cell proliferation at the molecular level. Functional evaluation by means of insulin secretion of the bioprinted xenograft have been demonstrated *in vitro* and *in ovo*. Rapid vascular ingrowth and neoangiogenesis can be found in all scaffold components as a premise for long-term function *in vivo.* On the one hand that the functional properties of the scaffold itself can be tuned at multiple levels and on the other hand that multicellular composition can further contribute to mimicking the natural microenvironment, thus improving functional outcomes.

This concept represents an intermediate step between approaches that require prevascularization of the implantation site^49^ and approaches in which host blood vessels are fused to bioartificially engineered graft vessels.^50^ We propose that an attachment encasing neurovascular structures might contribute to vessel formation while avoiding the instant blood-mediated inflammatory reactions that lead to graft loss, as described in clinical islet transplantation.^4^ Further *in vivo* studies on perivascular implantation of the simulation-optimized device and variations in its geometry (Figure 6(c), Supplementary Movie 4) are necessary. The parametric design as proposed in Figure 6 would allow variations of patient-specific devices that could be customized depending on, for example, the specific anatomy, and could overcome scalability concerns. Theoretically, building-blocks may be fused to form larger, multifunctional geometrical units augmenting the flexibility of tissue engineering applications. In the future, patients suffering from either type 1 or type 3c DM might benefit from this bioartificial insulin-secreting device by virtue of improved glycemic control, insulin independence, and quality of life.

## Supplementary Material

Supplementary material

Supplementary material

Supplemental Video 1

Supplemental Video 2

Supplemental Video 3

Supplemental Video 4
