# Supplementary material for: Toward 3D-bioprinting of an endocrine pancreas: A building-block concept for bioartificial insulin-secreting tissue
Source: J Tissue Eng. 2022 Apr 20;13:20417314221091033. doi: 10.1177/20417314221091033 (PMC9024162; doi:10.1177/20417314221091033)
Supplement: Supplementary material [file sj-docx-1-tej-10.1177_20417314221091033.docx]

# **Supplementary Information**

# **Supplementary Figure 1A**

# 3D-printed polycaprolactone component


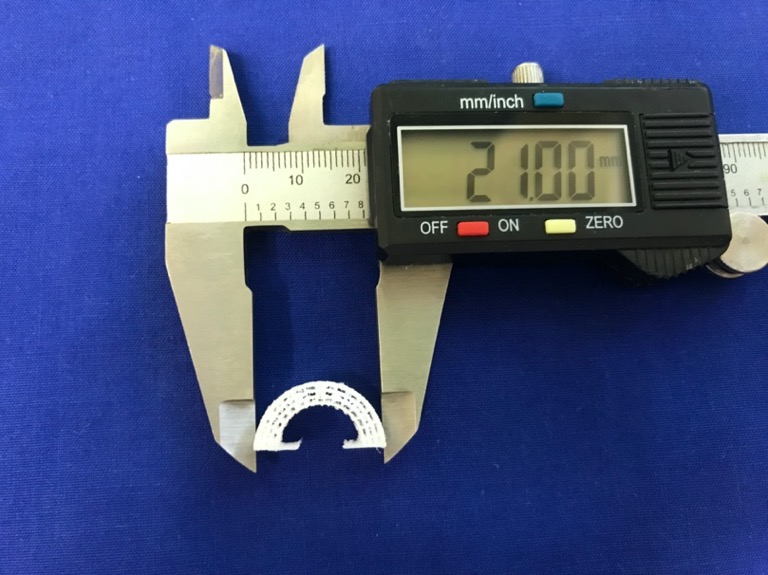


# **Supplementary Figure 1B**

HUVEC adhesion on heparinized PCL-scaffold. Scanning electron microscopy.


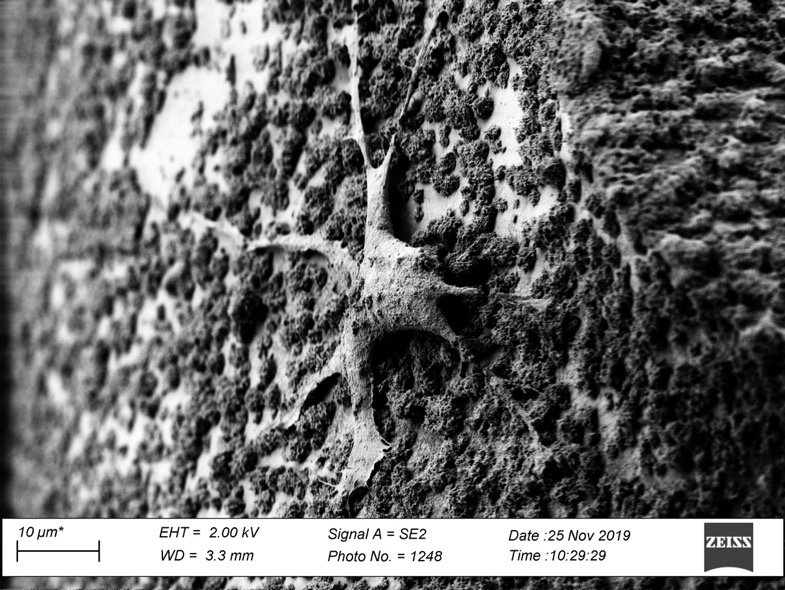


# **Supplementary Figure 1C**

# Glucose-stimulated insulin-secretion: INS-1 cells seeded on 3D-printed polymer scaffolds


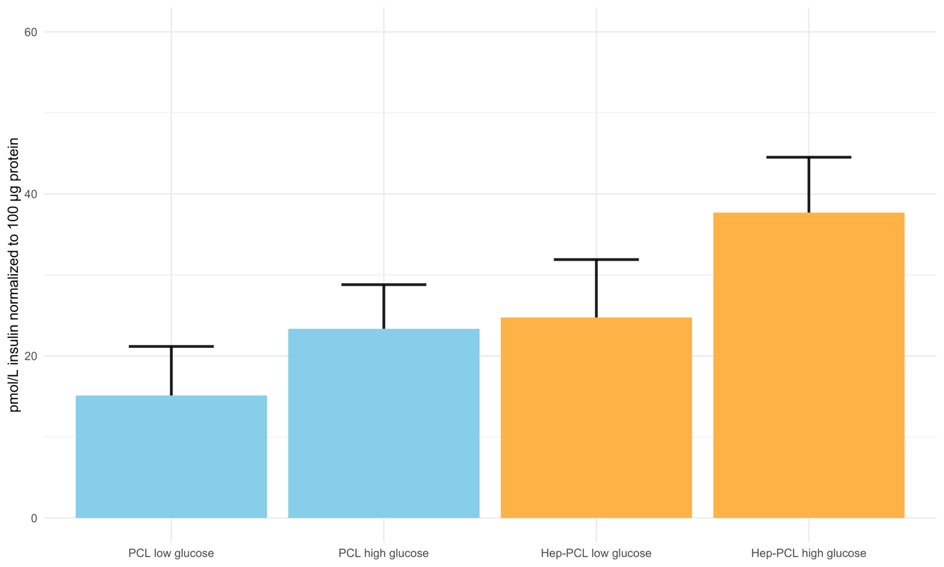


# **Supplementary Figure 2**

# 3D-bioprinted droplets: gelatin methacrylate blend / INS-1 cells

| 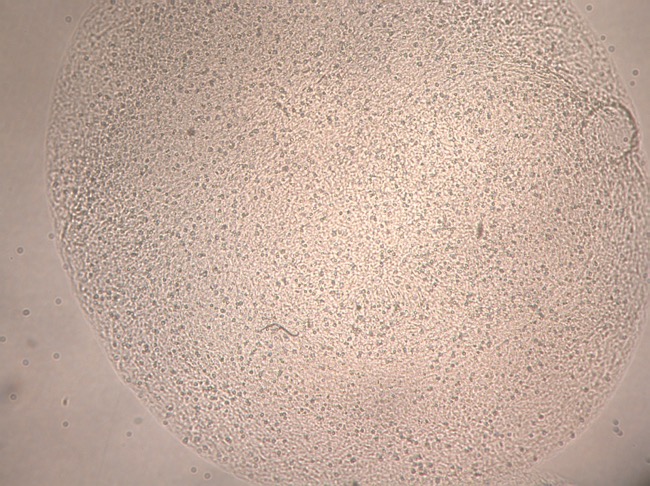 | Day 1 post-printing  INS-1 832/3 cells  CellInk GelXA LAMININK 411  Seeding density 2x10^6/ml  4x magnification |
| --- | --- |
| 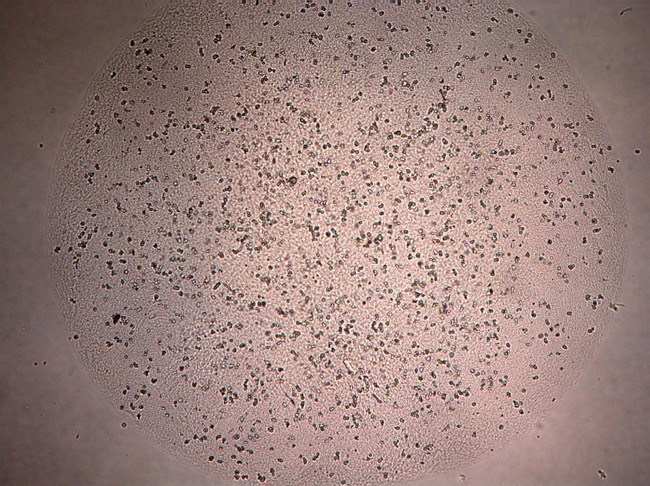 | Day 4 post-printing  INS-1 832/3 cells  CellInk GelXA LAMININK 411  Seeding density 2x10^6/ml  4x magnification |
| 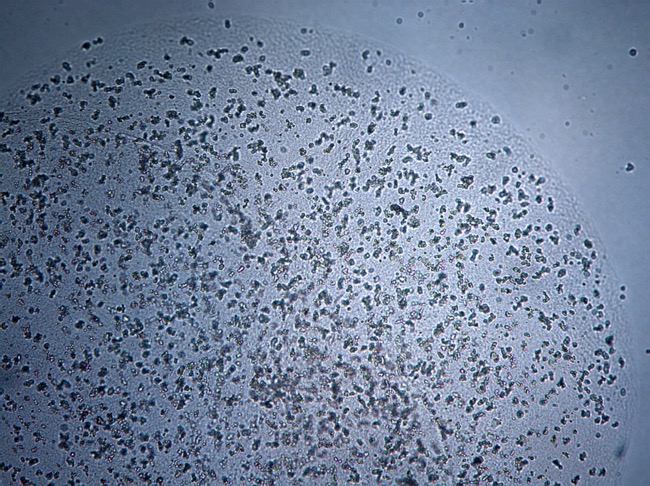 | Day 6 post-printing  INS-1 832/3 cells  CellInk GelXA LAMININK 411  Seeding density 2x10^6/ml  4x magnification |
| 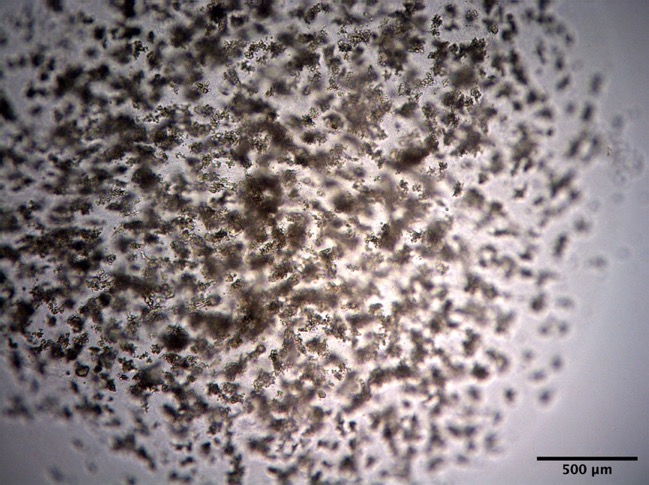 | Day 10 post-printing  INS-1 832/3 cells  CellInk GelXA LAMININK 411  Seeding density 2x10^6/ml  4x magnification |

# **Supplementary Figure 3**

# MTT assay staining – INS-1 cells encapsulated in hydrogel on day 7 post-bioprinting


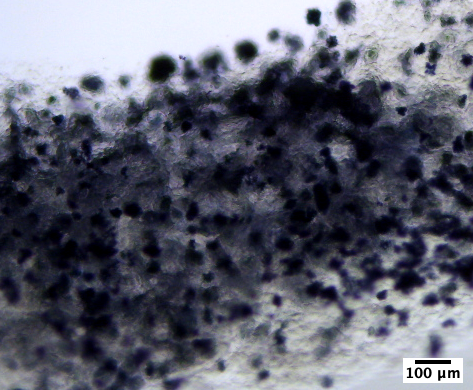


# **Supplementary Figure 4**

# **
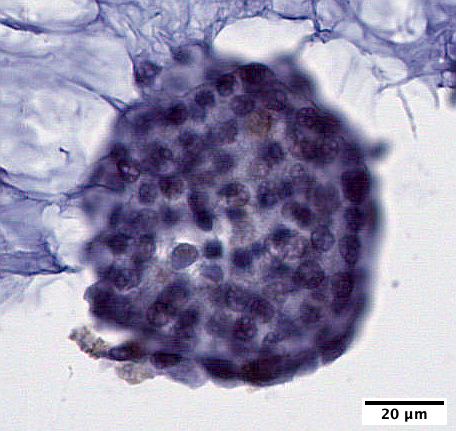

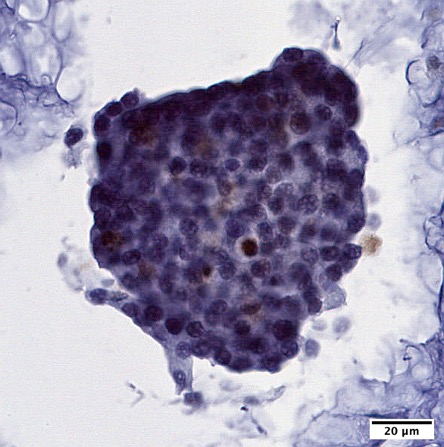

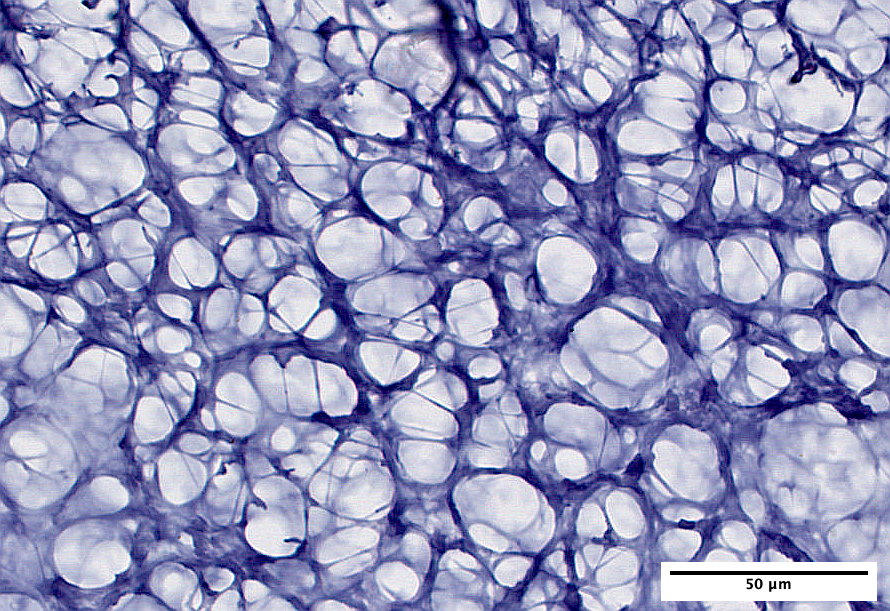
**Immunohistochemical staining of cleaved caspase-3 (brown, asterisk). Bioprinted INS-1 cells cultivated for 12 days.

# **
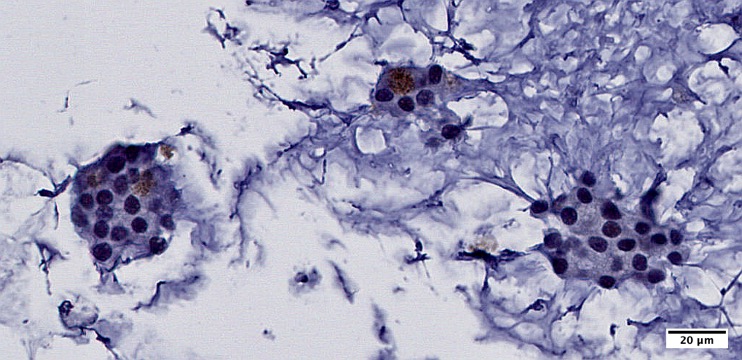

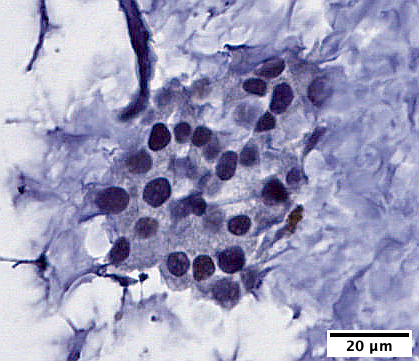
**

# **Supplementary Figure 5A**

Scanning electron microscopy of freeze-dried hydrogel structures (GelXA LAMININK 411) with different UV-curing times. Electron microscopy showed different morphological features of the hydrogel depending on the crosslinking time. 5A: 2s crosslinking


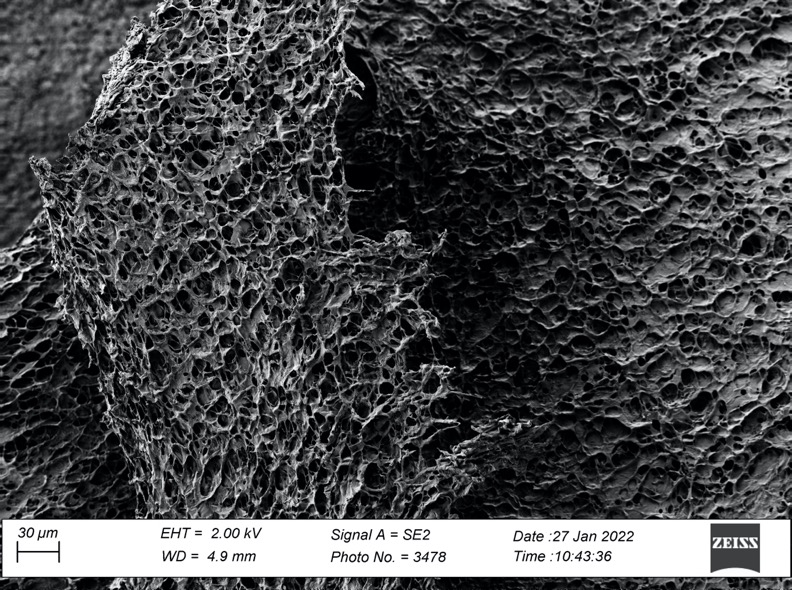


# **Supplementary Figure 5B**

Scanning electron microscopy of freeze-dried hydrogel structures (GelXA LAMININK 411) with different UV-curing times. Electron microscopy showed different morphological features of the hydrogel depending on the crosslinking time. 5A: 5s crosslinking


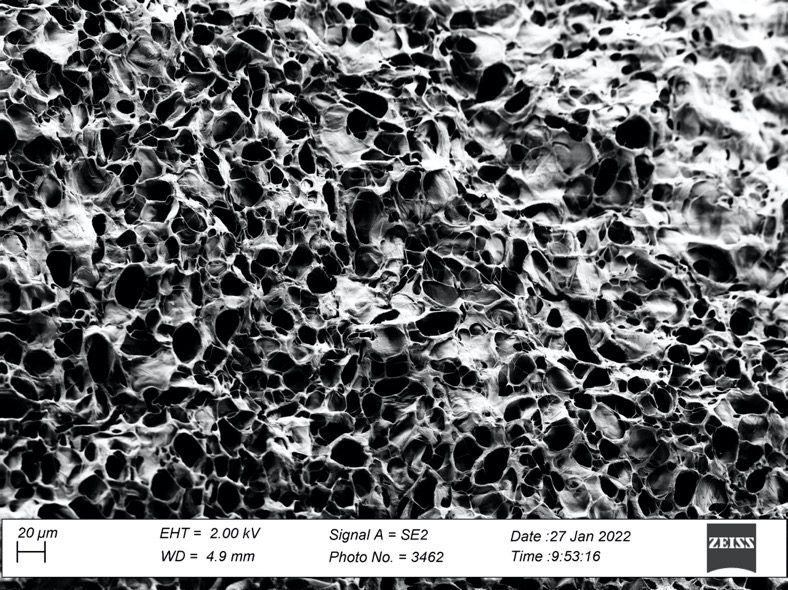


# **Supplementary Figure 5C**

Scanning electron microscopy of freeze-dried hydrogel structures (GelXA LAMININK 411) with different UV-curing times. Electron microscopy showed different morphological features of the hydrogel depending on the crosslinking time. 5A: 10s crosslinking


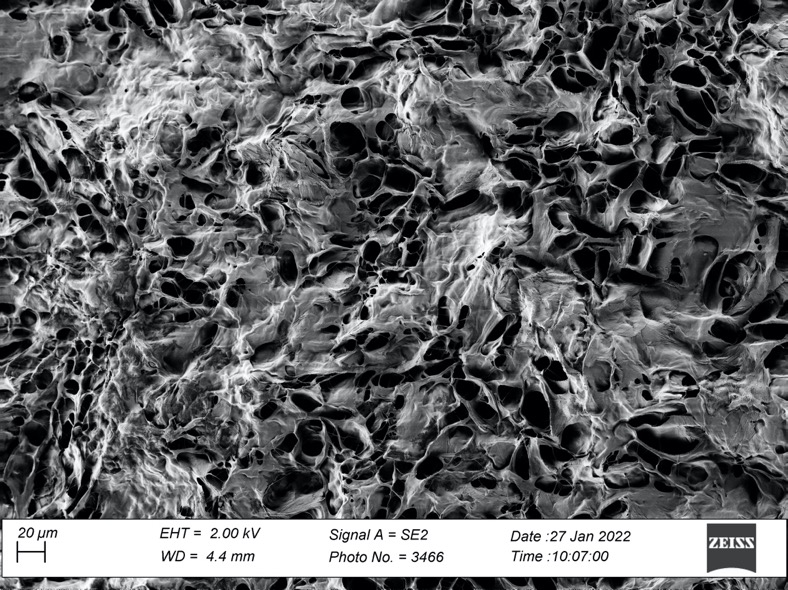


# **Supplementary Figure 6**

Diffusion assay- Diffusion of FITC-Dextran (50µg/ml). Time-dependent diffusion distance of hydrogels with different crosslinking times.

# **Supplementary Figure 7A**

# 3D-bioprinted grid structures incl. INS-1 for total RNA sequencing


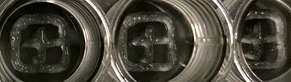


# **Supplementary Figure 7B**

# Principal component analysis total RNA sequencing


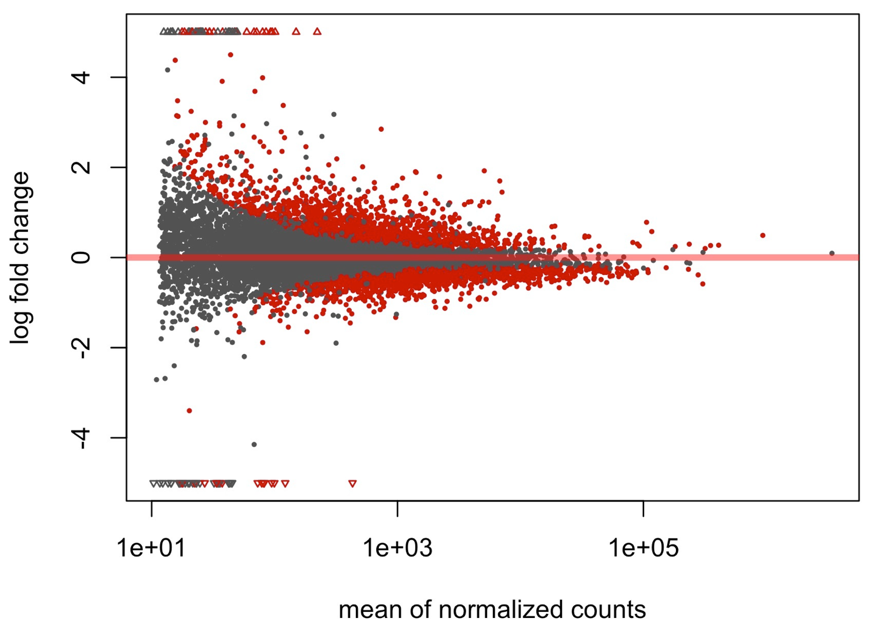


# **Supplementary Figure 7C**

# GSEA - hallmark pathways


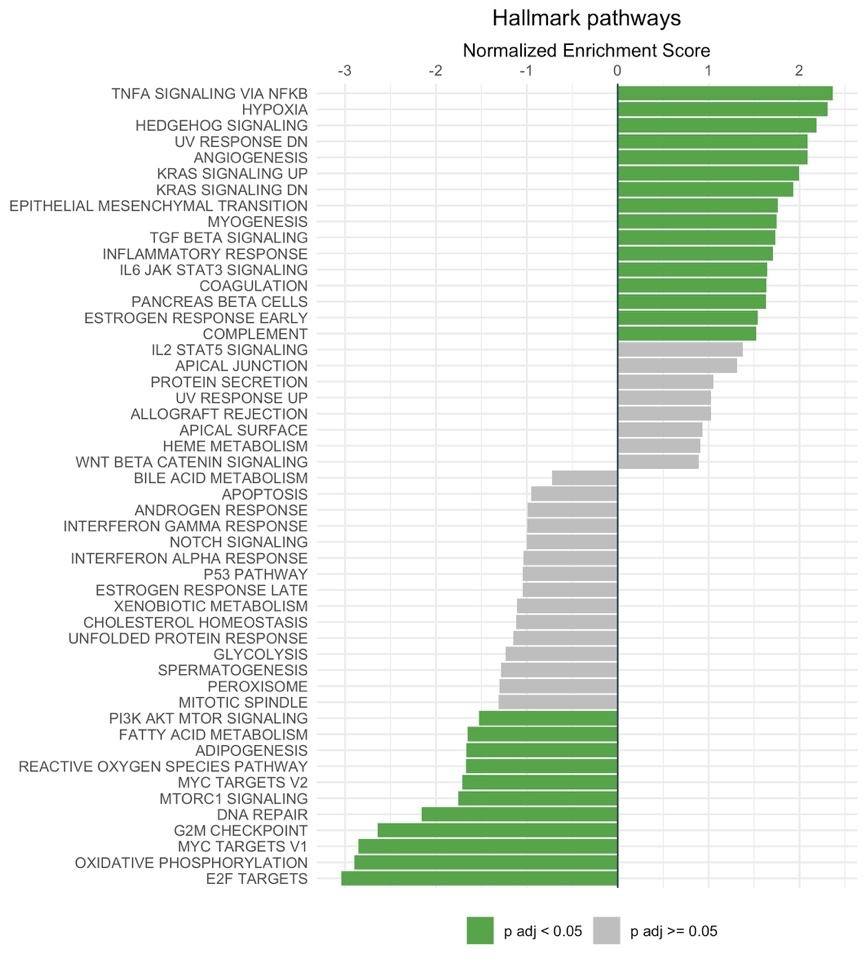


# **Supplementary Figure 7D**

# Ingenuity Pathway analysis – 100 altered canonical pathways (p≤0.05)


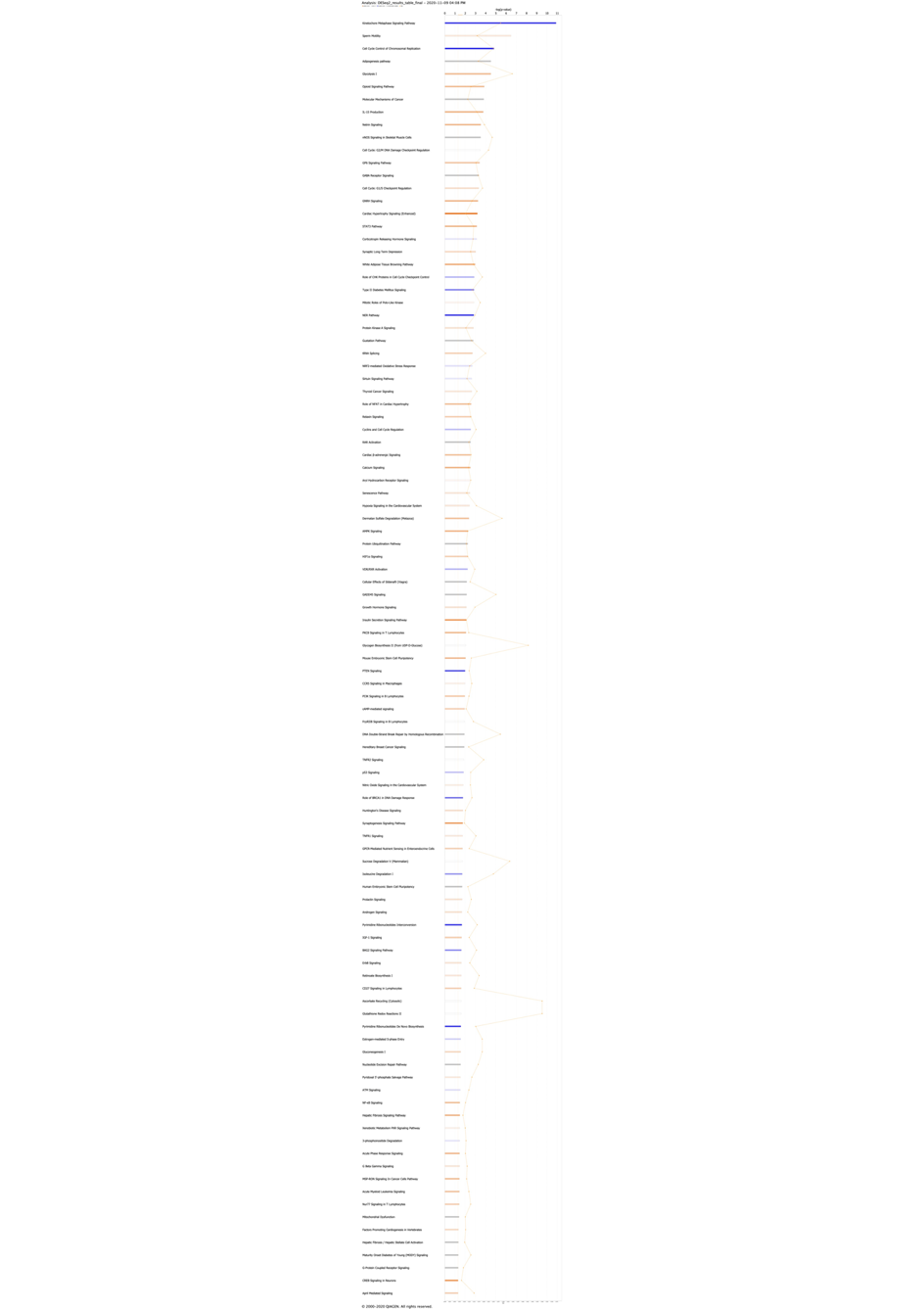


# **Supplementary Figure 7E**

# Ingenuity pathway analysis – graphical summary


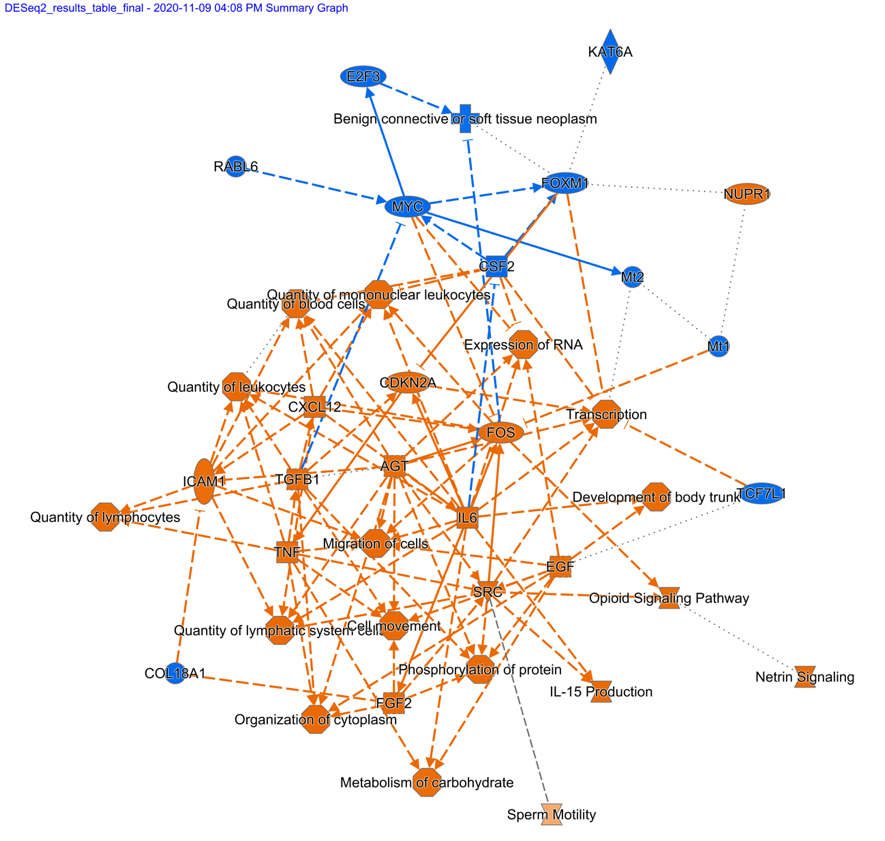


# **Supplementary Figure 7F**

# Ingenuity pathway analysis – upstream regulator analysis

Glucose as upstream regulator, data set genes, effect on target molecules and overlay of canonical pathways (insulin secretion signaling, insulin receptor signaling, glycolysis I, HIF1a signaling)

#
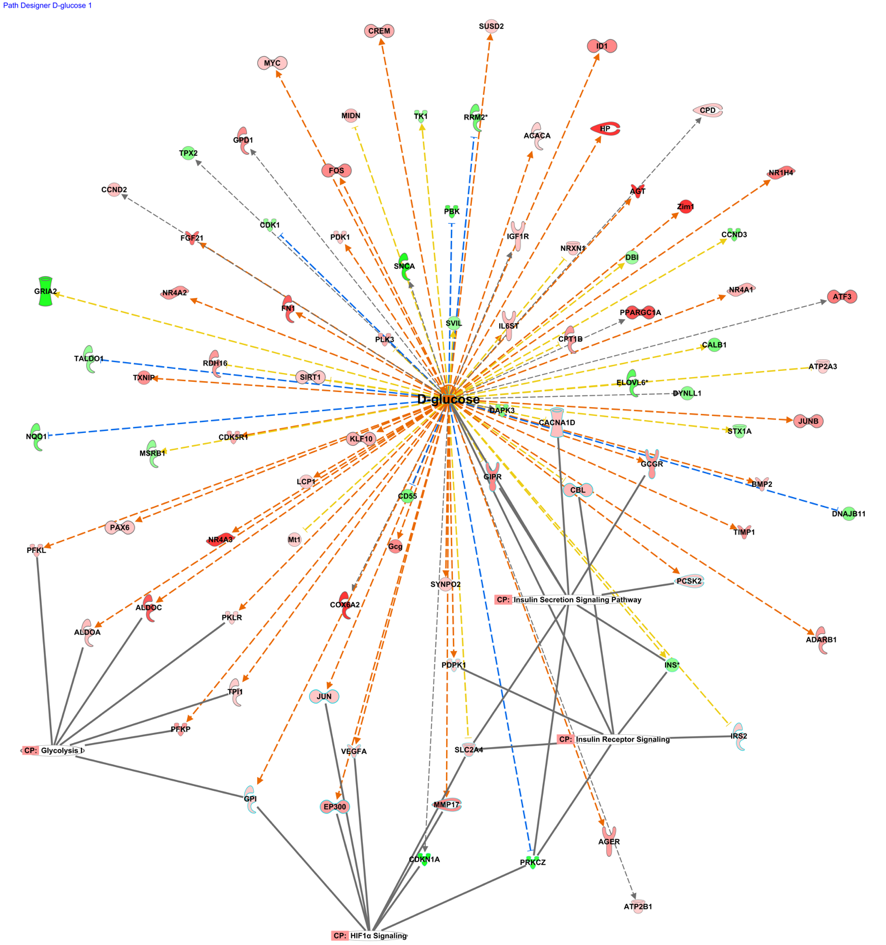


# **Supplementary Figure 7G**

# Exemplary table - differential gene expression

# **Supplementary Figure 8A**

# Vascular ingrowth and neoangiogenesis in scaffold structures

ML-based vascular network analysis: Explant of PCL scaffolds from CAM assay


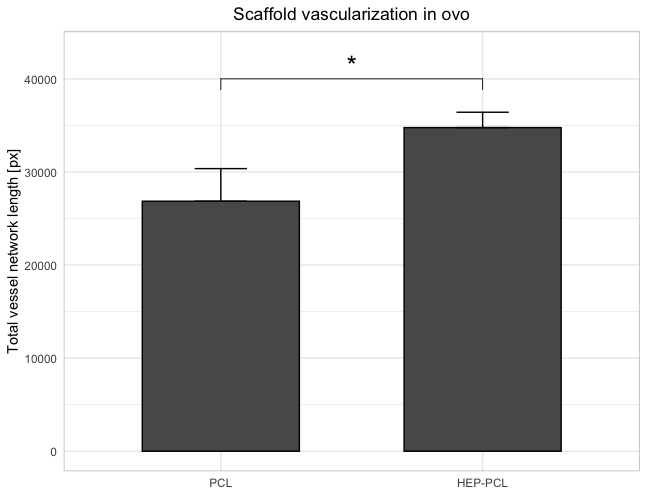


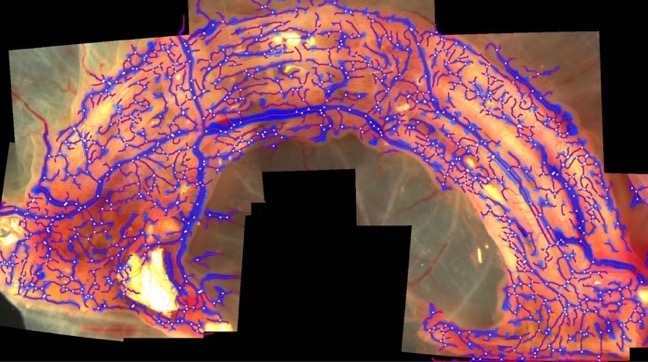


# **Supplementary Figure 8B**

# Chorioallantoic membrane assay – ex ovo trials, timeline

| 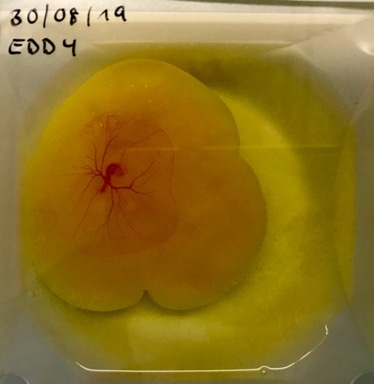 | **Embryonic development day (EDD) 4/18**  After 4 days of incubation, transfer of the viable chick embryo into culture device with glass top for observation |
| --- | --- |
| 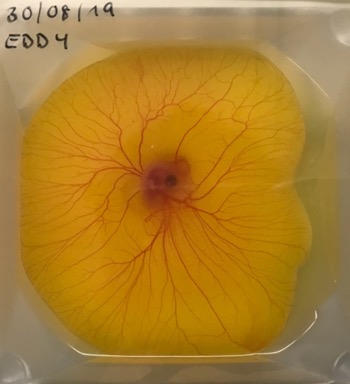 | **EDD 7/18**  Viable embryo |
| 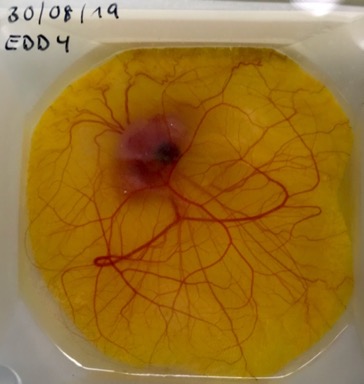 | **EDD 9/18**  Viable embryo, pre-implantation of solid polymer component scaffold |
| 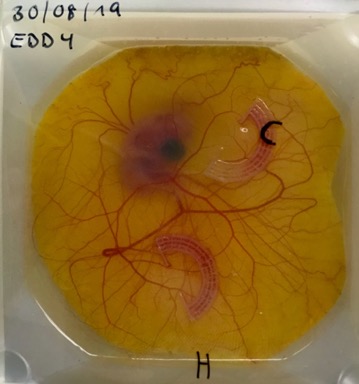 | **EDD 9/18**  Viable embryo, post-implantation of solid polymer component scaffolds  For direct comparison 3D-printed, sterilized untreated polycaprolactone scaffold and heparinized polycaprolactone were implanted. As described in the methods section, the. epithelial layer is gently scratched. (In case of the heparinized scaffold, the functionalization leads to a prolonged bleeding.) |
| 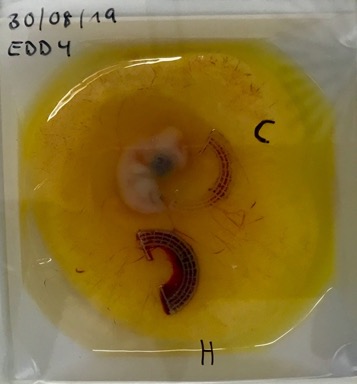 | **EDD 11/18**  Dead embryo, extensive bleeding around heparinized scaffold |

# **Supplementary Figure 8C**

# Immunohistochemical staining of CAM assay explant: gelatin methacrylate blend / INS-1 cells

Paraffin embedded tissue, slice thickness 5 µm, avian antiCD34 staining, bottom: periphery of xenograft, top: center of xenograft


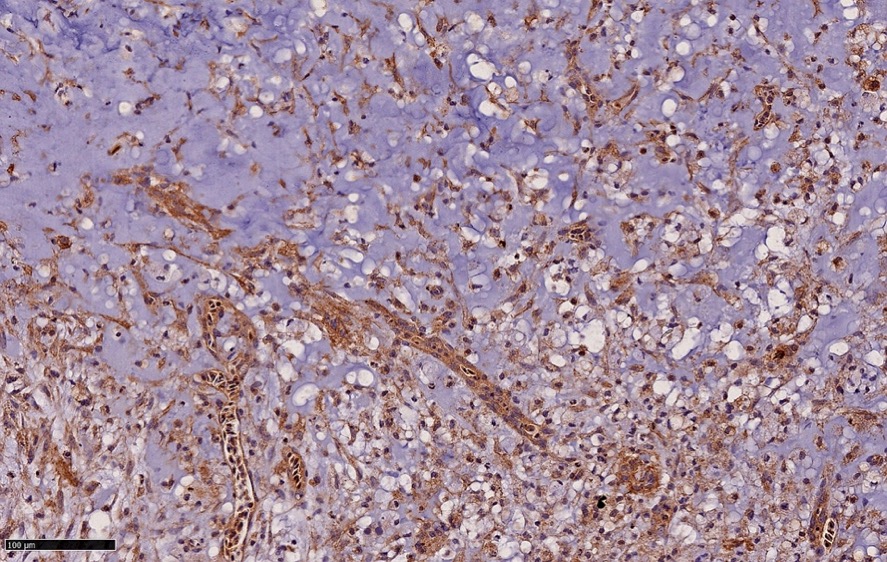


# **Supplementary Figure 9**

# Pixel Classification with ilastik – Exemplary data on spatial distribution of pseudoislets of cross-sections from bioprinted xenografts explanted from CAM (anti-insulin immunohistochemistry)

Pseudoislet segmentation (Insulin^+^ staining) using ilastik pixel classification with overlay of hydrogel graft area (1.3mm^2^, 1.8% pseudoislet area). Cross-section layer at 20µm (from graft bottom).

Pseudoislet segmentation (Insulin^+^ staining) using ilastik pixel classification with overlay of hydrogel graft area (1.8mm^2^, 2.1% pseudoislet area). Cross-section layer at 40µm (from graft bottom).



Pseudoislet segmentation (Insulin^+^ staining) using ilastik pixel classification with overlay of hydrogel graft area (6.5 mm^2^, 2.1% pseudoislet area). Cross-section layer at 60µm (from graft bottom).

Pseudoislet segmentation (Insulin^+^ staining) using ilastik pixel classification with overlay of hydrogel graft area (10 mm^2^, 0.1% pseudoislet area). Cross-section layer from xenograft with 15s UV-crosslinking (405nm) after bioprinting

Pseudoislet segmentation (Insulin^+^ staining) using ilastik pixel classification with overlay of hydrogel graft area (21.6 mm^2^, 0.6% pseudoislet area). Cross-section layer from large xenograft with 15s UV-crosslinking (405nm) after bioprinting

# **Supplementary Figure 10A**

# Glucose-stimulated insulin-secretion: experimental workflow

INS-1 cells were either growing in monolayer (2D samples) or embedded in bioprinted LAMININK 411 hydrogels (3D samples). To investigate the influence of endothelial cells on INS-1 cells, hydrogels containing a 1:2 co-culture of INS-1 and HUVEC were printed. Glucose stimulated insulin secretion (GSIS) was performed and cells were counted using fluorescence microscopy.

# **Supplementary Figure 10B**

# Glucose-stimulated insulin-secretion: culture conditions for INS-1 cells, 2D monolayer vs. 3D-hydrogel

#
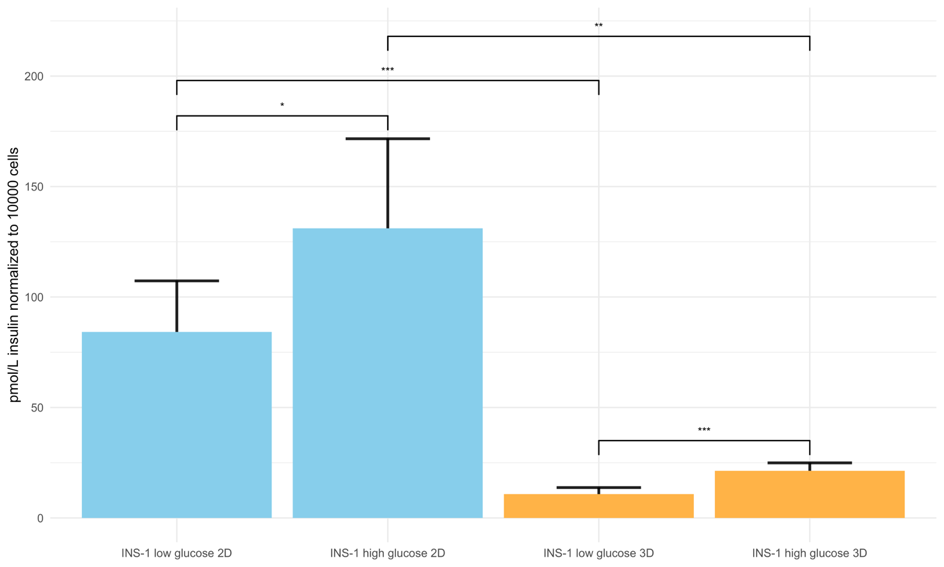


# **Supplementary Figure 11**

# In silico analysis: Parameters for computer-aided structure screening

| **Parameter** | **Value** |
| --- | --- |
| ***Oxygen*** |  |
| initial and inflow concentration | 0.1305 mol/m³ (equ. 90 mmHg) |
|  | 0.232 mol/m³ (equ. 160 mmHg) |
|  | 0.3915 mol/m³ (equ. 270 mmHg) |
| diffusion through aqueous media | 3.0 x 10^-9^ m²/s * |
| diffusion through hydrogel | 2.5 x 10^-9^ m²/s * |
| diffusion through Langerhans islet | 2.0 x 10^-9^ m²/s * |
| ***Glucose*** |  |
| initial and inflow concentration | 5 mol/m³ |
|  | 10 mol/m³ |
|  | 15 mol/m³ |
|  | 25 mol/m³ |
| diffusion through aqueous media | 9 x 10^-10^ m²/s * |
| diffusion through hydrogel | 6 x 10^-10^ m²/s * |
| diffusion through Langerhans islet | 3 x 10^-10^ m²/s * |
| ***Insulin*** |  |
| initial and inflow concentration | 0 mol/m³ |
| diffusion through aqueous media | 1.5 x 10^-10^ m²/s * |
| diffusion through hydrogel | 1.0 x 10^-10^ m²/s * |
| diffusion through Langerhans islet | 0.5 x 10^-10^ m²/s * |
| ***Islet of Langerhans*** |  |
| radius | 50 µm |
|  | 75 µm |
|  | 150 µm |
|  | 250 µm |
| ***Hydrogel*** |  |
| shell thickness | 0 µm |
|  | 50 µm |
|  | 100 µm |
|  | 300 µm |
|  | 500 µm |
|  | 600 µm |
|  | 700 µm |
|  | 800 µm |
|  | 1000 µm |

*As described by Buchwald. et al. (2011)
