# Supplementary figures and images for: Toward 3D-bioprinting of an endocrine pancreas: A building-block concept for bioartificial insulin-secreting tissue
Source: J Tissue Eng. 2022 Apr 20;13:20417314221091033. doi: 10.1177/20417314221091033 (PMC9024162; doi:10.1177/20417314221091033)

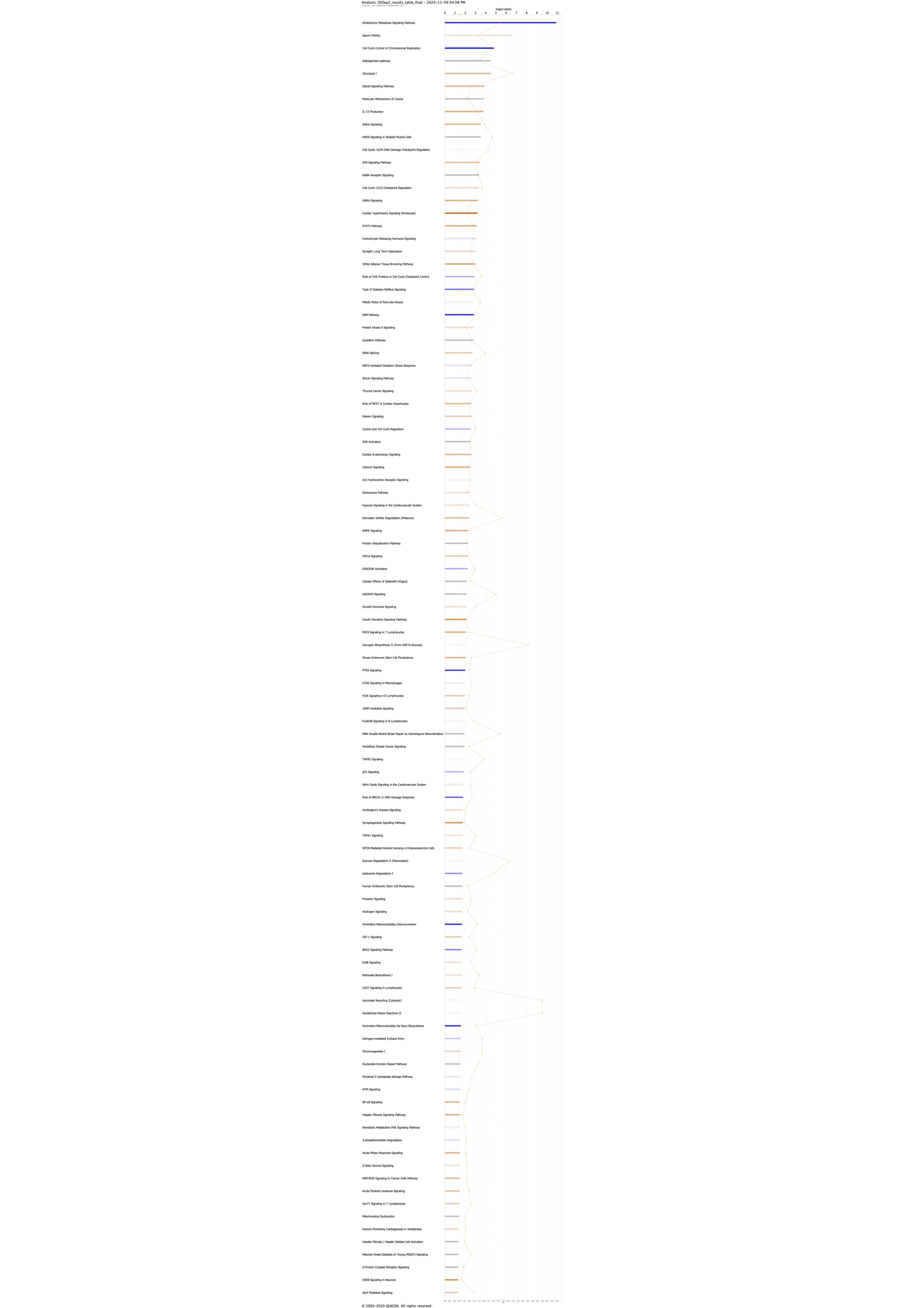

Supplement: Supplementary material [file sj-jpg-2-tej-10.1177_20417314221091033.jpg]
